# Getting Intentional about Intention to Use: A Scoping Review of Person‐Centered Measures of Demand

**DOI:** 10.1111/sifp.12182

**Published:** 2022-02-03

**Authors:** Victoria Boydell, Christine Galavotti

**Affiliations:** ^1^ School of Health and Social Care University of Essex Colchester UK; ^2^ The Bill and Melinda Gates Foundation Seattle WA 98109 USA

**Keywords:** intention to use, contraception, scoping review, demand

## Abstract

In recent years, there has been much reflection on the measures used to assess and monitor contraceptive programming outcomes. The meaning and measurement of intention‐to‐use (ITU) contraception, however, has had less attention and research despite its widespread inclusion in many major surveys. This paper takes a deeper look at the meaning and measurement of ITU around contraception. We conducted a scoping review guided by the following questions: What is the existing evidence regarding the measurement of ITU contraception? What definitions and measures are used? What do we know about the validity of these measures? We searched databases and found 112 papers to include in our review and combined this with a review of the survey instruments and behavioral theory. Our review found growing evidence around the construct of ITU in family planning programming and research. However there are inconsistencies in how ITU is defined and measured, and this tends not to be informed by advances in behavioral theory and research. Further work is needed to develop and test measures that capture the complexity of intention, examine how intention differently relates to longer‐range goals compared to more immediate implementation, and demonstrate a positive relationship between ITU and contraceptive use.

## INTRODUCTION

Understanding women's demand for contraception is essential for designing, implementing, and assessing responsive contraceptive programs. There are currently two ways to measure demand. The first is measuring women's unmet need for contraception, which is a top‐down population measure of need. The unmet need has been the main measure of demand since the 1970s, though it has undergone much critique and revision (Westoff 1978; Westoff and Ochoa 1991; Bradley et al. 2012).^1^^,^ 2 The second way to capture demand for family planning is to measure the intention‐to‐use (ITU) contraception, which has also been measured since the 1970s using items such as “I intended to do x.”^3^ Unlike unmet need, ITU draws on a woman's directly expressed desire to use contraception, her perception of risk to pregnancy, or her interest to use in the future (Ross and Winfrey 2001; Khan et al. 2015). This more person‐centered measure of demand directly captures women's stated preferences about using contraception and may actually better predict need and actual use (Ross and Winfrey 2001).

There have been advances in how we understand and measure intentions in the fields of social psychology and behavioral theory. Drawing on this work, we understand that intentions signal the end of a person's deliberation processes about what actions one will perform, how hard one will work for it, and how much effort one will apply to achieve the desired outcomes (Ajzen 1991; Gollwitzer 1993; Webb and Sheeran 2006; Gollwitzer and Sheeran 2008). Here, we use Triandis's (1980, 203) definition of intention: “Behavioral intentions are instructions that people give to themselves to behave in certain ways.” The nature of intention and its relationship to behavior has been theoretically mapped in a range of behavioral theories and models (e.g., the theory of planned behavior, self‐regulation theories, and phased models). Each theory has its own model of intention and its relationship to action. Whether the theory is based on goal striving theory of planned behavior, self‐regulation, or a staged model of behaviors (transtheoretical model, health belief model), they share a common understanding that intention is not a dichotomous variable, rather, it can be strong or weak, and it is conditioned, among other things, on time (i.e., intention to do immediately vs. at some point in the future) as well as proximity and attributes of the intended behavior. These advances in definition and measuring intention have yet to be applied to the construct of ITU in family planning.

In recent years as part of the post‐2012 Family Planning Summit renaissance, there has been much reflection on the measures used to assess and monitor contraceptive programming, for example, unmet need, additional users, demand satisfied, and continuation have been reexamined and refined (Cleland et al. 2014; Bradely and Casterline 2014; Dasgupta et al. 2017). Compared with other measures, ITU has received relatively little attention and it has not been subject to extensive independent research in the same way. The few scholars working on ITU argue that it merits further attention, concerning capturing ideational formation and demand for contraception and as a proximal predictor of future contraceptive use and it is time to get intentional about intent to use (Babalola et al. 2015; Hanson et al. 2015; Sarnak et al. 2020; Curtis and Westoff 1996; Callahan and Becker 2014). This scoping review presents the first attempt to synthesize what we know about ITU and hopes to start to fill this evidence gap. The following paper outlines the findings from a scoping review that examines the extent, range, and nature of the evidence on measuring ITU and the trends and gaps in the evidence with the aim of galvanizing research interest on this promising person‐centered measure of demand.

## METHODS

Our aim was to examine the extent, range, and nature of the evidence on measuring ITU. We chose a scoping review because it allowed us to survey a topic that has yet to be examined comprehensively. This review methodology allowed us to encompass a range of evidence sources and uses a variety of study selection approaches. Our research methodology is based on Colquhoun et al. (2014) framework for scoping reviews, which includes (1) identifying the research question; (2) identifying relevant studies; (3) study selection; (4) charting the data; and (5) collating, summarizing, and reporting the results (also see Arksey and O'Malley 2005; Levac et. al. 2010).

**FIGURE 1:**
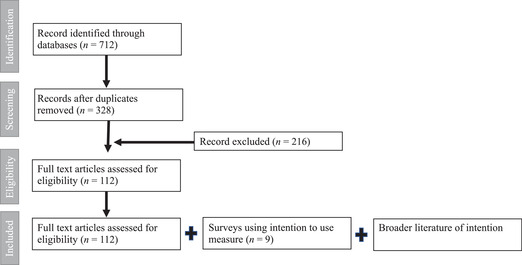
Flowchart of scoping review

The review was guided by the questions: What is the existing evidence regarding the measurement of ITU contraception? What definitions and measures are used? What do we know about the validity of these measures? To identify the relevant studies, we used three data sources: (1) we searched relevant databases of the peer‐reviewed literature, (2) we reviewed survey instruments that collected ITU, and (3) we reviewed the broader literature. Figure 1 outlines our scoping review process.

To identify the peer‐reviewed literature, we ran search strings using synonyms of “intent to use” and “contraception” in PubMed and Web of Science. The search terms included (intent^*^ OR intend^*^) AND (“to use”) OR (intent^*^ OR intend^*^ OR willingness) AND (contracept^*^ OR “birth control” OR “family planning”). A total of 712 records were found and downloaded into an excel spreadsheet. We reviewed the spreadsheet for duplicate records and 384 were removed. The titles and abstracts of the remaining 328 records were reviewed against the following inclusion criteria: refers a measure of ITU; refers any modern contraceptives, including condoms and emergency contraception; published between 1990 and present; and published in English. After the review, we retained 112 papers for full paper review (see Table A3 for bibliography).

A data‐charting form was developed that detailed the variables we extracted. We abstracted data on the following categories:

- Author;
- Year of publication;
- Country;
- Study design;
- Research objectives;
- Target population;
- Outcome of interest;
- Definition of ITU;
- Theoretical frameworks, if any;
- How ITU was measured;
- Findings.

We grouped the studies by the measures, theories, and the study design used and broad findings.

In addition to the peer‐reviewed sources of evidence, we also reviewed how different surveys measured ITU (see Table A1). To further contextualize the findings in the broader literature, we also read key papers on intentions from behavioral science. To provide a picture of the extent, range, and nature of existing evidence on ITU, we compare the findings from the peer‐reviewed search with those from two other data sources.

## RESULTS

Intention is a complex construct, and there is much debate about the nature of intention and if and how it relates to action. Behavioral theory distinguishes between types of intention, its properties, intensities, and determining variables. How we think intention works is central to how we operationalize it in measurement. After describing the characteristics of the evidence, in this section we detail the findings. The findings are grouped in relation to the definition of ITU, how the measure is used, the characteristics, and the reported results.

### Characteristics of the Evidence

The peer‐reviewed papers are described in Table 1, together with a description of the research objective and study design for each paper alongside details about the theoretical framework, the ITU items used, and the reported results.

**TABLE 1:** Details of included papers

Year	Author	Study Design	Research objectives	Theoretical framework	ITU item	Reported results
1993	A. Joffe and S. M. Radius USA	Cross‐sectional survey	Does self‐efficacy theory explain intent to use condoms of sexually and nonsexually active adolescents in the USA	Self‐efficacy theory	If you are planning to have sex, how likely is it that you will use a condom next time?	All eight behaviors of condoms were significantly correlated with intent to use condoms for males and six behaviors for females. The frequency of past condom use was most influential in explaining intent to use condoms for sexually active youths. Perceived self‐efficacy plays a modest role in predicting adolescent's intent to use condoms
1996	S. L. Curtis and C. F. Westoff MOROCCO.	Prospective Cohort Study Multivariate analysis	Assess the predictive effect of contraceptive intentions on subsequent individual contraceptive behavior in Morocco	Not given	“Do you intend to use a method to delay or avoid pregnancy at any time in the future?” “Do you intend to use a method within the next 12 months?”	Reported contraceptive intentions in 1992 have a strong predictive effect on subsequent contraceptive use even after controlling for other characteristics The strength of the effect is second only to that of the previous contraceptive Past use of contraceptives, spousal communication, and exposure to positive FP messages increase intent to practice.
1996	S. J. Hiltabiddle GLOBAL	Systematic review	To examine the factors associated with adolescent condom use	Health belief model	Not detailed	Health considerations played a minor role in determining condom use compared to other immediate consequences, such as perceived inconvenience of use and perceived partner attitude. Perceptions of individuals influence their health behavior: attitudes and beliefs, confidence, previous use, partner perception associate with intention‐to‐use (ITU)
1996	Minh Nguyet Nguyen, Jean François Saucier, and Lucille A. Pica CANADA	Cross‐sectional survey	To determine whether attitudes and other variables are associated with intention to use condoms in sexually inactive male adolescents in Canada	Theory of reasoned action	Not detailed	Information on condoms provided by peers was significantly associated with the intention to use condoms; information provided by schools, while not significant in the final model, was positively associated with younger adolescents' intention to use condoms; Sexually inactive male adolescents in a less committed couple relationship had a greater intention to use condoms
1999	E. Zimmers, G. Privette, R. H. Lowe, and F. Chappa USA	Randomly assigned field experiment	To assess the impact of viewing a video on women with a willingness to try and to continue using female condoms in the USA	Social psychological perspective	Not detailed	Although the video influenced women to try the product, it did not guarantee that they would be the device or continue to use it. Video viewing was unrelated to liking the product and future intent to use.
1999	Sara Ann Peterson, NIGER	Cross‐sectional survey Multivariate logistic regression	To examine the differences between polygamous and monogamous spouses with regard to approval of birth control, desired family size, communication between spouses, and intention of using birth control in Niger	Not given	Not detailed	The marital structure is not significantly associated with ITU birth control when the husband's age and the wife's ideal number of children were controlled Educational level and age at first marriage were significantly associated with attitudes towards birth control and also with marital structure.
2000	Marie Pierre Gagnon, and Gaston Godin USA	Randomized control trial with vignettes	To assess the potential effects of new antiretroviral therapies on preventive sexual behaviors by exploring the intention of young adults to maintain condom use following a modification in the outcomes of HIV infection	Theory of planned behavior and theory of interpersonal behavior	“If the occasion presents itself, I would use a condom with a new sexual partner”; “I would use a condom if I had a new sexual partner”; “I evaluate my chances of using a condom with a new sexual partner as …”	For this construct, a Cronbach's alpha coefficient of 0.87 was found and test–retest reliability coefficient was 0.99 Intention to use condoms with a new sexual partner is likely to be modified by the expected outcome of the disease, that is, whether lethal or chronic. New antiretroviral therapies are likely to modify the intention of college students to use a condom with a new sexual partner
2000	Ingela Lundin Kvalem and Bente Træen NORWAY	Randomized control trial	To examine the relationship between contraceptive self‐efficacy and the intention to use condoms among Norwegian adolescents	Self‐efficacy and sexual script theory	Not detailed	Connection between the adolescents’ actual use of condoms at the most recent sex and their intention to do so the next time Girls’ ITU at next sex was not influenced by their contraceptive self‐efficacy to the same degree as the boy's intention
2001	Anna Fazekas, Charlene Y. Senn, and David M. Ledgerwood CANADA	Cross‐sectional survey	To identify the variables that best predict whether or not young women intend to use condoms during their sexual encounters with new partners	Theory of planned behavior	Attitudes Toward Condoms Questionnaire (ATCQ) number of subscales tapping different dimensions of condom attitudes	Prediction was improved by including specific beliefs (condom use demonstrates responsible sexual activity, condom use does not destroy trust), group norms, and birth control use (mediated by attitudes toward condoms)
2001	John A. Ross and William L. Winfrey GLOBAL	Cross‐sectional survey	To assess what proportion of women during experience unmet need for contraception and what proportion express an intention to use and, second, how much do these women account for all unmet needs in an entire population, and how much do they represent all women who intend to practice contraception	Not given	• Do you intend to use a method to delay or avoid pregnancy in the next 12 months?	Over the first year postpartum, the proportion of women using a contraceptive rise, reducing the base of nonusers. As a result, the overall percentage intending to use declines with time, from 54% in the first three months postpartum to 31% in months 9–12) Where the stated intention to use contraceptives is high, actual use rises substantially On average, for each increase of 1% in intention, there is nearly a 1% rise in contraceptive adoption
2001	S. Agha ZAMBIA	Cross‐sectional survey	To examine intention to use the female condom among men and women in Zambia, who were exposed to mass‐marketing of the female condom	Not given	“Do you intend to use the female condom in the future?” “Do you intend to use the male condom in the future?”	In spite of a high level of awareness of the female condom, use of this method in the last year was lower than the use of the male condom Intention to use the female condom in the future was highest among respondents who had used only the female condom in the last year Discussion of the female condom with a partner was an important predictor of intention to use
2002	Anna Graham, Laurence Moore, Deborah Sharp, and Ian Diamond UK	Cluster randomized controlled trial	To assess the effectiveness of a teacher‐led intervention to improve teenagers’ knowledge about emergency contraception in the UK	Not given	Not detailed	The intervention and control groups did not differ in the proportion of pupils intending to use emergency contraception in the future
2002	Sohail Agha and Ronan Van Rossem TANZANIA	Cross‐sectional survey Path analysis is a regression based	To assess whether a mass media campaign designed to promote the use of the female condom had an impact on intentions to use the female condom among men and women of reproductive age in Tanzania	Diffusion of innovation	Not detailed	Mass media exposure significantly increased the likelihood that a man or a woman would discuss the use of the female condom with a partner. In turn, discussion of the female condom with a partner strongly influenced the intention to use the female condom in the future
2003	Michael R. Spence, Kindra K. Elgen, and Todd S. Harwell USA.	Cross‐sectional survey	To identify factors associated with awareness of emergency contraception (EC), prior use of EC, and intent to use EC in the future among women at the time of pregnancy testing in the USA	Not given	“Would you use emergency birth control?”	Women presenting for pregnancy testing would consider using it in the future (13%). Women who considered using EC in the future were more likely to be younger, non‐White, have <12 years of education, not currently living with a partner, and their usual source of care was a public clinic. Women who considered using EC in the future were also more likely to not want to be pregnant now or ever (21%) compared to women who wanted to be pregnant now or sooner (12%), or with those who were unsure of their current pregnancy (7%) Women with unintended pregnancies were no less likely to be aware of EC compared to women with intended pregnancies; however, they were more likely to consider using EC in the future
2003	Neeru Gupta, Charles Katende, and Ruth E. Bessinger UGANDA	Pre–Post Delivery of Improved Services for Health (DISH) evaluation surveys	To examine the associations between multimedia behavior, change communication (BCC) campaigns, and women's and men's use of and intention to use modern contraceptive methods in target areas of Uganda	Theory of diffusion of innovation		Exposure to BCC messages was associated with increased contraceptive use and intention to use in the near future for nonusers, regardless if they were men and women
2003	T. K. Roy, F. Ram, Parveen Nangia, Uma Saha, and Nizamuddin Khan INDIA	Cohort survey Logistic regression	To examine intention to use a method as a measure of contraceptive demand	Not given		Women who were not using a method (including those who were currently pregnant) were asked about their intentions to use a method and about their intended method
2003	I. Mogilevkina, and V. Odlind UKRAINE	Unmatched case‐control study	To investigate contraceptive practices and factors behind contraceptive preferences among Ukrainian women attending for abortion or gynecological health check‐up	Not given	Not detailed	A history of previous childbirth (odds ratio (OR) = 1.8), at least two induced abortions (OR = 1.7) and sexual education obtained from the literature (OR = 1.8) were associated with preference to use modern contraceptives
2004	Ruhul Amin, and Takanori Sato USA	Pre‐ and postintervention and comparison sites	To systematically evaluate a school‐based comprehensive program to assess its impact contraceptive use, future contraceptive intention	Not given	Not detailed	The percentages of enrolees in intervention schools who were using contraceptives or who expressed intention to use contraceptives in the future were higher than those of the counterpart nonenrolees from the comparison school
2004	Ruey Hsia Wang, Min Tao Hsu, and Hsiu Hung and Wang TAIWAN	Cross‐sectional survey adolescent boys	To explore the predictors of contraceptive intention in adolescent males in Taiwan	Theory of reasoned action (TRA) and self‐efficacy		Contraceptive intention was measured using a six‐item scale to assess the possibility of contraception use if a participant wanted to have sexual intercourse within the next year
2004	Margareta Larsson, Karin Eurenius, Ragnar Westerling, and Tanja Tydén SWEDEN	Quasi‐experimental, pre‐ and postintervention with a control logistic regression	To evaluate a community‐based intervention regarding emergency contraceptive pills (ECP), including a mass media campaign and information to women visiting family planning clinics	Theory of diffusion of Innovation and the health belief model		Intention to use emergency contraceptive pills in case of need was reported by 74% of the women and remained stable over time, but logistic regression showed that information during the previous year contributed to the willingness to use the method in the intervention group
2005	Sara J, Newmann, Alisa B. Goldberg, Rodolfo Aviles, Olga Molina de Perez, and Anne F. Foster‐Rosales EL SALVADOR	Cross‐sectional survey with postpartum adolescents	To describe demographics and contraceptive familiarity and use among postpartum adolescents in El Salvador	Not given	‘‘Do you plan on using contraception in the future?’’	Having a partner and living with him were predictors of intent to use contraception (*p* = 0.001 and 0.002, respectively). Being single negatively predicted intention to use contraception (*p* = 0.001)
2005	Cynthia Rosengard, Jennifer G. Clarke, Kristen DaSilva, Megan Hebert, Jennifer Rose, and Michael D. Stein USA	Cross‐sectional survey with women	To explore correlates of intentions to use condoms with main and casual partners among incarcerated women	Theory of planned behavior	“How often do you plan to use condoms in the next six months (following release from the Rhode Island Department of Corrections) with your [main/steady, casual/nonsteady] partner?”	Past behavior is often one of the best predictors of intentions regarding future behavior Condom use at last sex with a main partner, sexually transmitted disease (STD) history, no strong desire to currently be pregnant, belief that others influence one's health, and perceived STD risk was positively associated with women's intention to use condoms with main partners. Pregnancy history was negatively associated with intention to use condoms with the main partner. Condom use at last sex with a casual partner was positively associated with intention to use condoms with a casual partner
2005	Jennifer K. Legardy, Maurizio Macaluso, Lynn Artz, and Ilene Brill USA	Randomized control trial A binomial regression analysis	To assess whether participant baseline characteristics modified the effects of a skill‐based intervention promoting condom use	Not given	Not detailed	Intention to use condoms next time was a significant modifier of the intervention's effectiveness on consistent condom
2005	Daryl B. O'Connor, Eamonn Ferguson, and Rory C. O'Connor UK	Cross‐sectional survey	To explore the extent to which male hormonal contraception is perceived as risky compared to other prevention behavior s and examine the effects of message framing on intentions to use hormonal male contraception	Prospect theory The theory of planned behavior (TPB)	Not detailed	Exposure to a loss framing of messages influenced intention to use the daily male pill in men with a more positive attitude
2006	Ekere James Essien, Gbadebo O. Ogungbade, Harrison N. Kamiru, Ernest Ekong, Doriel Ward, and Laurens Holmes NIGERIA	Cross‐sectional study	To examine multiple predictors of condom, use among Uniformed Services Personnel in Africa in Nigeria	Self‐efficacy theory	Respondents were asked if there is a particular place, they go to get a condom.	The knowledge of how to correctly wear a condom was the most significant positive predictor of the intention to use a condom, adjusted prevalence odds ratio (APOR), 5.99, (95% CI = 1.26, 19.79). Other main positive predictors of intent to use condoms were the knowledge of the mode of HIV transmission via blood, APOR 2.43 (95% CI = 1.01, 5.82), saliva (5.87, 95% CI 3.15, 10.94), and preejaculatory fluid (APOR, 3.58, 95% CI = 1.67, 7.48). Male gender was also a significant positive predictor of the intent to use condoms, APOR, 2.55, (95% CI 1.10, 5.97) Compared to men, women were of disadvantage in intent to use condoms; Monogamous relation negatively predicted the intent to use condoms
2006	Cees Hoefnagels, Harm J. Hospers, Clemens Hosman, Leo Schouten, and Herman Schaalma NETHERLANDS	Cross‐sectional survey.	To investigate whether ITU condoms change when there is no pregnancy risk, to allow such changes to be predicted from an STD risk‐perception perspective with undergraduate students in the Netherlands	Not given	Not detailed	Intention not to use a condom was three times as high (58 vs. 19) when there was no risk of pregnancy compared to the condition in which no information about contraception was provided. Thirty‐seven percent of the respondents stated that they intended to use a condom with a new sexual partner when there was no risk of pregnancy. The different types of risk (pregnancy and STD risk) were associated with the distinction between consistent intenders (who intended to use a condom irrespective of birth control issues) and nonconsistent intenders who in‐ tended to use a condom when the birth control issue was implicit, but did not intend to use a condom when there was no risk of pregnancy)
2006	Hong Ha M. Truong, Timothy Kellogg, Willi McFarland, Mi Suk Kang, Philip Darney, and Eleanor A. Drey USA	Examination of medical records of adolescents	To examine the changes between a choice of contraceptive methods before abortion and contraceptive intentions after abortion in the USA	Not given	Analysis of medical records—individual change in primary contraceptive choices before abortion compared to contraceptive intentions after abortion	There was no difference in intention to use condoms after abortion among adolescents who received voluntary HIV counseling and testing compared to those who did not
2007	Eun Seok Cha, Kevin H. Kim, and Willa M. Doswell KOREA	Cross‐sectional survey	This study examined the mediating role of condom self‐efficacy between the parent‐adolescent relationship and the intention to use condoms in Korea	Theory of reasoned action	Not detailed	Condom self‐efficacy mediated the prediction of intention to use condoms by the quality of the mother‐son relationship
2008	Ruey‐Hsia Wang, Chung‐Ping Cheng, and Fan‐Hao Chou TAIWAN	Cross‐sectional survey	To investigate how social influences, attitude, and self‐efficacy operate together to influence contraceptive intention in Taiwan	Theory of reasoned action	“If you want to have sex within the next year, how strong is the possibility of your using contraceptives?”	Social influences affected contraceptive intention indirectly through the contraceptive attitude and self‐efficacy for contraception, which affected contraceptive intention directly
2008	Cynthia J, Mollen, Frances K. Barg, Katie L. Hayes, Marah Gotcsik, Nakeisha M. Blades, and Donald F. Schwarz USA	Qualitative research	To explore the knowledge, attitudes, and beliefs of urban, minority adolescent girls about intention to use emergency contraception pills and to identify barriers to emergency contraception pill use among 15–19 Black adolescent girls in hospital settings in the USA	Theory of planned behavior	If they would be considering ECP in the future	Urban, minority adolescent girls have misconceptions about ECPs are affected by the opinions of those close to them, and express concern about specific barriers
2008	M. Williams, A. Bowen, M. Ross, S. Timpson, U. Pallonen, and C. Amos USA	Cross‐sectional survey Two structural equation models	To investigate the contribution of a personal norm of condom‐use responsibility to the formation of intentions to use male condoms during vaginal sex among heterosexual African American crack cocaine smokers in Houston, Texas	Integrated behavior al model	Respondents were asked if s/he intended to use a male condom the next time s/he had vaginal sex with the named partner	A personal norm of condom‐use responsibility provided a significantly better fit to the data than models that only included outcome expectations, subjective norms, and self‐efficacy A personal norm of condom‐use responsibility had a strong direct effect on men's intentions to use condoms with the last sex partner. Women's intentions were strongly influenced by a personal norm and social subjective norms Strong direct paths in men's and women's models from a personal norm of condom‐use responsibility to condom‐use self‐efficacy beliefs, subjective norms, and condom‐use attitudes suggest that developing a personal norm of condom use may be a necessary process before condom‐use attitudes, feelings of self‐ efficacy or subjective norms begin to change
2008	Hee Sun Kang, and Linda Moneyham KOREA	Cross‐sectional survey	To examine the intentions, knowledge, and attitudes of college students regarding the use of emergency contraceptive pills (ECPs) and condom in Korea	Theory of planned action	Not detailed	The intentions of using ECPs and condoms were positively correlated with each other and with a positive attitude. There were significant gender differences on many of the variables, in that female students had higher knowledge about ECPs, intention of using ECPs and condoms, and more positive attitudes toward condoms than male students who had more positive attitudes toward ECPs
2009	Omololu Adegbola, and AdeyemI Okunowo NIGERIA	Cross‐sectional survey	To assess the intention to use postpartum contraceptives and factors influencing use among pregnant and puerperal women in Lagos University Teaching Hospital (LUTH), Lagos, Nigeria	Not given	Not detailed	Advanced age and high parity significantly predicted intention to use postpartum contraceptives (*p* = 0.02 and 0.01, respectively). A high level of respondent's education and FP counseling by doctors and nurses increased the intention to use postpartum contraceptives (*p* = 0.03 and 0.01, respectively)
2009	G Anita Heeren, John B. Jemmott, Andrew Mandeya, and Joanne C. Tyler SOUTH AFRICA	Prospective cohort study	To test the hypothesis that hedonistic and normative beliefs regarding sexual partners and peers, and control beliefs regarding condom‐use technical skill and impulse control would predict the intention to use condoms, which would predict condom use three months later among undergraduates at a university in Eastern Cape Province, South Africa	Theory of planned behavior	Not detailed	Condom‐use intention predicted consistent condom use and condom use during most recent intercourse at three‐month follow‐up The hedonistic behavioral belief that condoms do not interfere with sexual enjoyment, normative beliefs that sexual partners and peers approve of condom use, and control beliefs reflecting confidence in condom‐use technical skill and the ability to exercise sufficient impulse control to use condoms were significantly related to the intention to use condoms This prospective intention–behavior link is held across two different ways of operationalizing the use of condoms—consistent condom use and condom use during most recent intercourse
2010	Sohail Agha PAKISTAN	Cross‐sectional survey	To assess the perceived costs and motivations for specific contraceptive use behaviors among currently married women 15–49 and men married to women 15–49 in Pakistan	Synthesis framework	“Do you or your spouse intend to use this method in the next 12 months?”	For women, the perception of in‐law's support, belief in birth spacing, perception method choice, and staff competency were drivers of intentions to use contraceptive methods. The strongest obstacle to ITU was a belief that family planning decisions were made by the husband, and fertility was determined by God's will. For men, the perception that FP improved the family and wife well‐being was the most important drivers of ITU. The strongest obstacle to ITU was a fear that contraceptives would make a woman sterile and harm her womb lowered his intention to use modern contraceptive methods
2011	Tina R Raine, Anne Foster‐Rosales, Ushma D. Upadhyay, Cherrie B. Boyer, Beth A. Brown, Abby Sokoloff, and Cynthia C. Harper USA	A 12‐month longitudinal cohort study	To assess contraceptive discontinuation, switching, factors associated with method discontinuation, and pregnancy among women initiating hormonal contraceptives among adolescent girls and women aged 15–24 years attending public family planning clinics, USA	Theory of reasoned action	“How sure are you that you will use the baseline method selected for 1 year?”	The only factors associated with a lower risk of discontinuation were greater intent to use the method Intention was guided by attitudes toward contraception, pregnancy desire, and partner's attitudes
2011	Saima Hamid, Rob Stephenson, and Birgitta Rubenson PAKISTAN	Cross‐sectional survey	To explore how young married women's involvement in the arrangements surrounding their marriage is associated with their ability to negotiate sexual and reproductive health decisions in marriage in Pakistan	Self‐efficacy theory	Not detailed	Having a say in the selection of a spouse was significantly associated with an agreement with the spouse over the number of children to have, ITU contraceptives, and the time between marriage and first contraceptive use The odds of ITU contraceptives were higher among respondents aged 20‐=24 years at marriage as compared to 15–19 years old The more children the respondents had, the higher was the likelihood of discussing and agreeing with their spouse on the number of children to have, ITU, or be current users Higher mobility outside the household was associated with higher ITU
2011	Katherine E. Brown, Keith M. Hurst, and Madelynne A. Arden UK	Randomized Control Trials	To evaluate the impact of intervention materials, designed to enhance self‐efficacy and anticipated regret, on contraceptive behavior and antecedents of contraceptive use in a sample of adolescents in the UK	Theory of planned behavior	‘‘I intend to use a method of contraception effectively every time I have sex’’	Among sexually active participants with relatively low levels of intention to use contraception at the outset, increases in several outcome measures including intention and behavior were observed (*F*[3,35] 1⁄4 10.359, P 5 0.001, Z2p 1⁄4 0.47) The finding that intervention conditions perform no better than control
2011	Jongwon Lee, Mary Ann Jezewski, Yow Wu Bill Wu, and Mauricio Carvallo USA	Cross‐sectional survey	To explore the relationship between acculturation and beliefs, attitudes, norms, and intention regarding oral contraceptive use among Korean immigrant women using acculturation in the USA	Theory of reasoned action	Intention to use oral contraceptives in the future	Acculturation affects intention to use oral contraceptives indirectly only through other components (e.g., changes in beliefs, attitudes, norms, and intention)
2011	V. Khanal, C. Joshi, D. Neupane, and R. Karkee NEPAL	Cross‐sectional survey	To find perceptions, practices, and factors affecting the use of family planning among abortion clients in Nepal		Intention to use FP after abortion	Knowledge, acceptance of counseling service, and intention to use family planning measure was high in the study participants
2012	Joy Noel Baumgartner, Rose Otieno‐Masaba, Mark A. Weaver, Thomas W. Grey, and Heidi W. Reynolds KENYA	Cross‐sectional survey	To explore the facility‐and provider‐level characteristics that may be associated with same day uptake or intention to use contraception after a Voluntary counseling and testing (VCT) visit, and contraceptive use three months later among youth clients in Kenya	Not given	Not detailed	For the outcome of same day uptake of FP or intention to use FP after the VCT session, significantly associated client‐level variables included having a partner (more so for those not living with their partner), having more education, having more children, and not wanting children in the next three months. There were no significant provider characteristics, but at the clinic level, higher levels of integration were significantly associated with higher FP uptake/intention to use
2012	Elizabeth Rink, Kris FourStar, Jarrett Medicine Elk, Rebecca Dick, Lacey Jewett, and Dionne Gesink USA	In‐depth interviews	This study examines the extent to which age, fatherhood, relationship status, self‐control of birth control method, and the use of birth control influence young Native American men's intention to use family planning services in the USA	Not given	“How likely is it that you will seek birth control services in the next year?”	Men with children were over five times more likely to seek FP services within the next year for birth control than Native American men who reported not having children
2012	Elizabeth Rink, Kris FourStar, Jarrett Medicine Elk, Rebecca Dick, Lacey Jewett, and Dionne Gesink USA	In‐depth interviews	To examine the influence of age, fatherhood, and mental health factors related to historical trauma and loss on young American Indian (AI) men's intention to use birth control in the USA	Not given	Not detailed	Fatherhood may also be considered a protective factor that may increase the likelihood that young Native American men will seek family planning services for birth control
2012	Shelly Campo, Natoshia M. Askelson, Erica L. Spies, and Mary Losch USA	Cross‐sectional survey.	To examine how the extended parallel process model (EPPM) constructs of fear, susceptibility, severity, response efficacy, and self‐efficacy related to young adult women's intention to use contraceptives	EPPM	‘‘How likely are you to use birth control the next time you have sexual intercourse?’’.	Perceived severity of the consequences of unintended pregnancy (p 5 0.01), communication with friends (p 5 0.01) and last sexual partner (p 5 0.05), relationship status (p 5 0.01), and past use (p 5 0.001) were associated with women's intentions to use contraceptives. Talking with their last sexual partner had a positive effect on intentions to use contraceptives while talking with friends influenced intentions in a negative direction Women who perceive an unintended pregnancy as more severe are more likely to intend to use contraceptives
2012	Catherine Potard, Robert Courtois, Mathieu Le Samedy, B. Mestre, M. J. Barakat, and Christian Réveillère FRANCE	Cross‐sectional survey	To identify the determinants of the intention to use and actual use of condoms in a sample of French adolescents based on Ajzen's theory of planned behavior with adolescent boys and girls in France	Theory of planned behavior	I intend to use a condom every time I shall have sex with a new partner in the next three months	“Intention”’ to use a condom during every sexual encounter was explained by “perceived control” and “individual attitudes”, and to a lesser extent by “subjective norms of close friends and relatives” and “socio‐cultural norms”
2012	Jin Yan, Joseph T.F. Lau,; Hi Yi Tsui, Jing Gu, and Zixin Wang CHINA	Cross‐sectional survey	To investigate the prevalence of male condom use and associated factors among monogamous STI female patients	Health belief model	Not detailed	Significant factors associated with lower likelihoods of consistent condom use in the past two months were type of sole sex partner (cohabitant vs. husband: OR = 0.29, 95% CI = 0.12. 0.70; regular boyfriend vs. husband: OR = 0.52, 95% CI = 0.30, 0.91), being financially dependent (OR = 0.45, 95% CI = 0.27, 0.75), and partner's dislike of condom use (OR = 0.23, 95% CI = 0.15, 0.39)
2013	Victor Akelo, Sonali Girde, Craig B. Borkowf, Frank Angira, Kevin Achola, Richard Lando, Lisa A. Mills, Timothy K. Thomas, and Shirley Lee Lecher KENYA	Cross‐sectional survey	To analyze FP attitudes among HIV‐infected pregnant women enrolled in a Prevention of mother‐to‐child transmission (PMTCT) clinical trial among HIV positive pregnant women about future FP use in Western Kenya	Not given	Intention to use any form of FP in the future	A significant gap exists between future FP intentions and current FP practices. Factors associated with positive intentions to use FP were: marital status (*p* = 0.04), having talked to their spouse or partner about FP (*p* = 0.001), perceived spouse or partner approval of FP (*p* = 0.001), previous use of a FP method (*p* = 0.006), attitude toward the current pregnancy (*p* = 0.02), disclosure of a sexually transmitted infection (STI) diagnosis (*p* = 0.03) and ethnic group (*p* = 0.03)
2013	Patrizia Di Giacomo, Alessia Sbarlati, Annamaria Bagnasco, and Loredana Sasso ITALY	Cross‐sectional survey	To describe what puerperal women know about postpartum contraception and to identify their related needs and expectations in Italy	Not given	Intention to use contraception in the postpartum period	During pregnancy and postpartum, 45·5% of the women reported that they had received adequate information about contraception. Of these ones, 64·3% reported their intention to use contraception either to avoid pregnancy or to space out future births Women's intention to use contraception was proportional to their level of education
2013	Rajesh Kumar Rai, and Sayeed Unisa INDIA	Cross‐sectional survey	To examine the reasons for not using any method of contraception as well as reasons for not using modern methods of contraception, and factors associated with the future intention to use different types of contraceptives in India	Not given	Not detailed	There is considerable variation in explaining the factors associated with future intention to use contraceptives
2014	Bernice Kuang, John Ross, and Elizabeth Leahy Madsen GLOBAL	Cross‐sectional survey	To define high and low motivation groups by stated intention to use, past use, and unmet need, to determine how these groups differ in characteristics and in the region of residence in the African continent	Not given	“Do you think you will use a contraceptive method to delay or avoid pregnancy at any time in the future?”	Nonusers were more rural, less educated, and closer to poverty and less likely to have access to public services. Intenders were younger and with smaller families. Also, their ideal family sizes are much smaller than those of nonintenders (though still high) Highly motivated nonusers, the most likely women to adopt contraception in the future, since they both intend to use and report past use
2014	Laili Irani, Ilene S. Speizer, and Jean‐Christophe Fotso KENYA	Cross‐sectional survey	To examine the association between relationship‐level characteristics (desire for another child, communication about the desired number of children, and FP use) and contraceptive use and intention to use among nonusers in Kenya		Social ecological theory	Not given
2014	Charles Picavet, Ineke van der Vlugt, and Ciel Wijsen THE NETHERLANDS	Cross‐sectional survey Multivariate analysis	To examine whether increased knowledge about ECPs may increase the intention to use these products among women in the Netherlands	Theory of reasoned action	Imagine you had intercourse without using contraception. You do not want to become pregnant. Would you take emergency contraception?	Intention is most strongly related to prior use and not having children; being aware that ECPs can be obtained without prescription upgrades intention, whereas knowing that a woman can still get pregnant after having taken the ECP has a negative impact on intention. Ambivalence concerning pregnancy is common and related to an unwillingness to use contraception
2014	Susan Krenn, Lisa Cobb, Stella Babalola, Mojisola Odeku, and Bola Kusemiju NIGERIA	Pre and post Cross‐sectional survey	To describe the activities designed and implemented by Nigerian Urban Reproductive Health Initiative (NUHRI) to meet the project's stated objectives and to illustrate how having a demand lens influenced programming decisions in ways that other family planning programs probably would not have considered in Nigeria	Ideation theory	Not detailed	Greater exposure to a comprehensive family planning program in urban Nigeria that emphasized demand generation and communication theory was associated with improved ideation among women (their beliefs, ideas, and feelings about family planning), and more positive ideation was associated with greater intention to use FP
2014	Getachew Mekonnen, Fikre Enquselassie, Gezahegn Tesfaye, and Agumasie Semahegn ETHIOPIA	Cross‐sectional survey	To assess the prevalence and associated factors of long‐acting and permanent contraceptive methods in Jinka town, southern Ethiopia	Not given	Not detailed	Knowledge of contraceptives and the age of women have a significant association with the use of long‐acting and permanent contraceptive methods Different variables, 90 (53%) of illiterate, 51 (64.6%) of those who can read and write, 276 (68.3%) of those with modern education, 57 (58.2%) of those with a college education have intention to use long‐acting and permanent methods Participants who perceive their social status to be very poor, poor, medium, and rich have intention to use long‐acting and permanent methods were 7 (58.3%), 111 (55.8%), 334 (65.4%), and 13 (68.4%) respectively
2014	Allahna Esber, Randi E. Foraker, Maryam Hemed, and Alison Norris TANZANIA	Cross‐sectional survey Multivariable logistic regression	To study the effect of partner approval of contraception on intention to use contraception among women obtaining post abortion care in Zanzibar, Tanzania	Not given	“Do you think you will use a method of contra‐ ception in the future?”	Adjusting for past contraception use, partner approval of contraception was associated with 20 times the odds of intending to use contraception (odds ratio, 20.25; 95% CI, 8.45, 48.56).
2014	Maricianah Onono, Cinthia Blat, Sondra Miles, Rachel Steinfeld, Pauline Wekesa, Elizabeth A. Bukusi, Kevin Owuor, Daniel Grossman, Craig R. Cohen, and Sara J. Newmann KENYA	Pre‐ and postintervention survey	To determine if a health talk on family planning (FP) by community clinic health assistants (CCHAs) will improve knowledge, attitudes, and behavioral intentions about contraception in HIV‐infected individuals in Kenya	The Information Motivation and Behavioral framework	Intention to initiate a new FP method	Following the health talk and clinic visit, 45% female participants and 33% of all participants reported they wanted to try a new FP method. Among females, the decision to try a new method was more commonly made by those who were using no method or only condoms than those using a more effective form of FP, (59% vs. 11%, *p* < 0.02). Among males, the proportion that decided they or their partner would try a new method did not differ by FP method use prior to the health talk (33% vs. 33%, *p* = 1.0)
2014	Md Mosfequr Rahman, Md Golam Mostofa, and Md Aminul Hoque BANGLADESH	Cross‐sectional survey	Explores women's decision‐making autonomy as a potential indicator of the use of contraception in Bangladesh	Not given	Not detailed	Household decision‐making autonomy is significantly associated with the current use of modern contraception, future intention to use contraception, and discussing contraception with the husband
2014	Alem Gebremariam and Adamu Addissie ETHIOPIA	Cross‐sectional study	To assess intention to use long‐acting and permanent contraceptive methods (LAPMs) and identify associated factors among currently married women in Adigrat town, Ethiopia	Not given	Not detailed	LAPMs were higher among women who knew at least one of LAPMs (AOR = 4.7, 95% CI = 1.58, 14.01) and women who do not want to have birth within the next 2 years (AOR = 1.9, 95% CI = 1.22, 3.13). Intention to use LAPMs was less among women who perceive poor support from their husbands (AOR = 0.2, 95% CI = 0.09, 0.45) and those who perceive LAPMs are harmful for the womb (AOR = 0.24, 95% CI = 0.14, 0.41). Similarly, participants in the focus group discussion have expressed their concern on the return of fertility after using implants or IUCD as well as insertion and removal procedures. The most preferred LAPMs were implants (71.3%). The most preferred LAPMs were implants (71.3%)
2014	Mengistu Meskele and Wubegzier Mekonnen ETHIOPIA	Cross‐sectional survey In‐depth interviews	To examine the association between women's awareness, attitude, and barriers with their intention to use LAPMs among users of short‐term methods in Ethiopia	Not given	Not detailed	Positive attitude, absence of myths, and misconceptions on LAPMs and secondary and plus a level of education predict intention to use LAPMs
2015	Tina R. Raine‐Bennett, and Corinne H. Rocca USA	Psychometric evaluation	To develop and validate the psychometric properties of the Contraceptive Intent Questionnaire (CIQ), an instrument that can enable providers to identify young women who may be at risk of contraceptive nonadherence in the USA	Theory of reasoned action	Measures the latent construct of contraceptive intent and is designed to capture both conscious and unconscious factors that comprise a woman's predisposition to use contraception developed items	The Contraceptive Intent Questionnaire (CIQ). CIQ has modest reliability and good internal validity. The separation reliability of the 15‐item scale of 0.73 met minimal acceptable standards
2015	Hae Won Kim KOREA	Cross‐sectional study with	To compare the ECP awareness of males and females and its associations with intention to use four other contraceptive methods (condoms, oral contraceptive pills, and withdrawal and rhythm methods) of unmarried university students in Korea	Theory of planned behavior	I will choose this method myself; I will use this method consistently; I will choose this method without another's recommendation	ECP awareness was associated with the intentions of students to use withdrawal or rhythm methods The multiple assessments of four kinds of contraceptive intentions were made at a time that differs from previous studies. Accordingly, the comparisons of the current study's findings to other findings could be given consideration
2015	Mishal S. Khan, Farah Naz Hashmani, Owais Ahmed, Minaal Khan, Sajjad Ahmed, Shershah Syed, and Fahad Qazi PAKISTAN	Unmatched case‐control study	To quantitatively evaluate the effect of family members' opposition to family planning on intention to use contraception among poor women who have physical access to family planning services in two public hospitals in Karachi, Pakistan	Not given	Information on women's intention to use contraception in the future was solicited by first mentioning the local names of a list of 11 contraceptive methods and asking (one by one) if the woman was aware of it (knowledge of contraception assessment) and then asking whether they intend to use any form of contraception in the near or distant future	Negative contraceptive intent was associated with no knowledge of contraception (AOR = 3.79 [2.43‐5.90]; p ∖ 0.001), husband's opposition (AOR = 21.87 [13.21‐36.21]; p ∖1) and mother‐in‐law's opposition (AOR = 4.06 [1.77‐9.30]; p ∖ 0.001). Husband's opposition has the strongest effect on women's intention to use contraception, even when the women have knowledge of and physical access to family planning services There is little evidence of an independent effect of parity on contraceptive intent
2015	Jongwon Lee, Mauricio Carvallo, and Taehun Lee USA	Psychometric evaluation	To evaluate the psychometric properties of a measure of attitudes and subjective norms toward OC use among Korean American women in the USA	The theory of reasoned	Not detailed	The measure and its use for determining the degree to which Korean immigrant women intend to use OCs is valid and reliable. Subjective norms (*r* 1⁄4 .56) and general attitude (*r* 1⁄4 .44) exhibited strong associations with intention to use OCs, whereas effective‐ ness (*r* 1⁄4 .24) and usability (*r* 1⁄4 .21) exhibited small associations with intention to use OCs The analysis revealed that subjective norms were a much stronger predictor of intention to use OCs (b 1⁄4 0.47, *p* < 0.001) than general attitude (b 1⁄4 0.26, *p* < 0.001). Even when women's OC use history was controlled for, the relative importance of subjective norms (b 1⁄4 0.45) over general attitude (b 1⁄4 0.27) did not differ
2015	Sadaf Khan, Breanne Grady, and Sara Tifft GLOBAL	Cross‐sectional survey	To describe a demand estimation exercise conducted in response to an initiative to introduce Sayana Press in 12 countries in Sub‐Saharan Africa and South Asia		Not given	
2015	Echezona E. Ezeanolue, Juliet Iwelunmor, Ibitola Asaolu, Michael C. Obiefune, Chinenye O. Ezeanolue, Alice Osuji, Amaka G. Ogidi, Aaron T. Hunt, Dina Patel, and Wei Yang, John E. Ehiri NIGERIA	Cross‐sectional survey	To determine if (1) male partners’ awareness of, and support for, female contraceptive methods, and (2) influence of male partners’ contraceptive awareness and support on pregnant women's expressed desire to use contraception among pregnant women and their male partners in Nigeria	Not given	Male participants were asked the following questions among others: (a) are you aware of types of female contraceptive methods? (b) If yes, mention any methods that you are aware of. (c) Would you support your spouse's use of any form of contraception (men's support for contraception)? (d) If yes, what type? Female participants were asked the following: (a) Are you aware of types of female contraceptive methods? (b) Are you interested in using any contraceptive method? (c) If yes, which type(s)?	Men's awareness of, and support for, use of modern contraceptives was significantly associated with their female partners’ desire to use contraception. A majority of the men who were aware of modern contraceptives. In addition, men who showed support for their spouses’ use of contraception were over five times more likely to have spouses who indicated a desire to use contraception (AOR = 5.76, 95% CI = 4.82, 6.88)
2015	Stella Babalola, Neetu John, Bolanle Ajao, and Ilene S. Speizer KENYA AND NIGERIA	Cross‐sectional survey	To examine differences and commonalities in the determinants of contraceptive use intentions in these two contexts with a special focus on ideational variables in Kenya and Nigeria	Ideation model of strategic communication and behavior change	Not detailed	The data revealed four dimensions of contraceptive ideation in both countries: perceived self‐efficacy, myths, and rumors related to contraceptives, social interactions and influence, and contraceptive awareness. All four dimensions of ideation are strongly associated with contraceptive use intention in Nigeria. Both perceived self‐efficacy and myths and rumors dimensions were significantly associated with contraceptive use intention in Kenya. In contrast, social interaction dimension and contraceptive awareness were not strongly associated with intention in Kenya. Striking similarities in the dimensions of ideation and the relationship between ideation and contraceptive use intentions in the two countries. The importance of self‐efficacy for contraceptive use intention in both countries is The odds of contraceptive use intention increase with parity may suggest that women in both countries tend to wait until they have a specific number of children before considering contraception.
2015	Ramos Mboane, Madhav P. Bhatta MOZAMBIQUE	Cross‐sectional survey	To examine the influence of a husband/partner's healthcare decision‐making power on a woman's intention to use contraceptives in Mozambique	Not given	Are you thinking about using any contraceptive method to delay or avoid getting pregnant in the future?	A husband/partner's healthcare decision‐making power in the relationship had a significant negative effect on a Mozambican woman's intention to use contraceptives.
2015	Jessica D. Hanson, Faryle Nothwher, Jingzhen Ginger Yang, and P. Romitti USA	Cross‐sectional survey	To test and analyze direct and indirect measures of perceived behavioral control (PBC) in the context of birth control use among women. In addition, it aimed to examine the associations between PBC measures, intention, and actual birth control use in the USA	The theory of planned behavior		Participants indicated a high level of control over using birth control, and a significant positive correlation was observed between direct and indirect PBC measures. Participants also reported high intentions to use birth control, and a significant positive correlation was observed between intention and PBC. Both PBC measures and intention were independently and significantly associated with behavior, and PBC remained significantly associated with behavior when intention was added into the model. In conclusion, compared to the previous literature, this study is unique in that it examines indirect PBC measures and also the important role that PBC plays with actual birth control behavior
2015	Fand Zavier and Shireen J. Jejeebhoy INDIA	Cross‐sectional survey	To better understand the contraceptive practices of young abortionseekers aged 15–24 years in India	Not given	Not detailed	Similar proportions (16%–19%) of unmarried and married adolescents had practiced contraception at the time of both their first and last sexual encounter, and that while many more intended to practice contraception postabortion, significantly fewer unmarried than married young women intended to do so (42% vs. 57%). ITU was significantly associated with the nature of sexual relations and the abortion experience, regardless of marital status The odds that an abortion‐seeker had intended to use contraception postabortion were greater among those who had practiced contraception earlier in their sexual life (odds ratios (OR), 2.6 among all respondents; 2.9 among the unmarried; 2.2 among the married), and among the unmarried, decreased among those who had experienced forced sex (OR, 0.5). Many reported their intention to practice postabortion contraception, more among married than unmarried young women
2015	Christopher Godwin Udomboso, A. Y. Amoateng. and P. T. Doegah GHANA and NIGERIA	Cross‐sectional study	To examine the effects of selected bio‐social factors on the intention to use contraception among never married and ever‐married women in Ghana and Nigeria	Not given	DHS	Intention to use is influenced by a range of social and biological factors Educational attainment, exposure to media, and visitation to a health facility affected intention to use contraception significantly and positively in both countries. On the other hand, number of living children, infrequent sexual intercourse, postpartum amenorrhea, opposition to contraception, and lack of access to contraceptives negatively affected intention to use contraception
2016	Yonatan Moges Mesfin and Kelemu Tilahun Kibret ETHIOPIA	Systematic review and meta‐analysis	To summarize the evidence of practice and intention to use long‐acting and permanent family planning methods among women in Ethiopia	Not given	Not detailed	Women's intention to use LAPCMs is generally good but their utilization is low prevalence of intention was 42.98% (95% CI 32.53, 53.27%). compared to practice was 16.64% (95% CI 12.4, 20.87%).
2016	Matthew F. Reeves, Qiuhong Zhao, Gina M. Secura, and Jeffrey F. Peipert USA	Prospective cohort study	To compare unintended pregnancy rates by the initially chosen contraceptive method after counseling to traditional contraceptive effectiveness in the same study population in the USA	Not given	Not detailed	Though highly effective in the as‐used analysis, women initially choosing injectable contraception had pregnancy rates similar to oral contraception and significantly worse than IUC or implantable contraception
2016	Jennifer H. Tang, Dawn M. Kopp, Gretchen S. Stuart, Michele O'Shea, Christopher C. Stanley, Mina C. Hosseinipour William C. Miller, Mwawi Mwale, Stephen Kaliti, Phylos Bonongwe and, Nora E. Rosenberg MALAWI	Prospective cohort study	To evaluate if implant knowledge and intent to use implant were associated with implant uptake of postpartum women in Malawi	Not given	The contraceptive methods she was planning to use in the first year after de‐ livery	Correct implant knowledge (adjusted HR = 1.69; 95% CI 1.06, 2.68) and intent to use implant (adjusted HR 1.95; 95% CI 1.28, 2.98) were both associated with implant uptake, with a stronger association for intent. The association between intent to use implant and implant uptake was stronger than the association between correct implant knowledge and implant uptake
2016	Kebede Haile, Meresa Gebremedhin, Haileselasie Berhane, Tirhas Gebremedhin, Alem Abraha, Negassie Berhe, Tewodros Haile, Goitom Gigar, and Yonas Girma ETHIOPIA	Cross‐sectional study	To assess magnitude and factors associated with a desire for birth spacing for at least two years or limiting childbearing and nonuse of LAPMs among married women of reproductive age in Ethiopia	Not given	Not detailed	Intention to use LAPMs were significantly associated with not using LAPMs
2016	Gareth Roderique‐Davies, Christine McKnight, Bev John, Susan Faulkner, Deborah Lancastle UK	Cross‐sectional survey	To investigate women's intention to use long‐acting reversible contraception using two established models of health behavior in the UK	The theory of planned behavior and the health belief model	Not detailed	The theory of planned behavior and the health belief model accounted for 75% of the variance in intention to use. Perceived behavior al control, perceived barriers, and health motivation predicts the use of long‐acting reversible contraception All multiitem constructs were shown to have internal consistency with all scoring over 0.8 The construct with the strongest predictive power was perceived benefits. The next strongest predictor variable in this model was the subjective norm in that the salience of the beliefs of others will increase or decrease a woman's intention to use LARC Intention was a significant predictor of whether a woman was using LARC
2016	Maureen French, Alexandra Albanese, and Dana R. Gossett USA	Retrospective cohort study Regression analysis	To evaluate the effect of high‐risk pregnancy status on antepartum contraceptive planning and postpartum use among women delivering at a university hospital in the USA	Not given	Not detailed	Low‐risk and high‐risk women showed interest in tier 1 contraceptive while antepartum (54.4% low‐risk vs. 58.0% high‐risk, p = 0.2), with lower interest at discharge (42.3% vs. 50.7%, *p* = 0.001) and the postpartum visit (33.8% vs. 40.1%, *p* = 0.002), while women with a high‐risk pregnancy were more likely than low‐risk women to indicate an intent to use highly effective methods, this did not translate into actual administration High‐risk women had similar rates of planning for tier 1 contraceptive but similar rates of subsequent unplanned pregnancy. Intention to use highly effective contraception did not translate into actual use In our regression analysis, factors predicting plans to use highly effective contraception in the postpartum period included Hispanic ethnicity and public insurance. public insurance was also a significant predictor of subsequent pregnancy, representing a failure in the actual use of such methods
2016	Fentanesh Nibret Tiruneh, Kun Yang Chuang, Peter A. M. Ntenda, and Ying Chih Chuang ETHIOPIA	Cross‐sectional survey	To explore the relationship of various predisposing, enabling, and need factors with the intention to use contraceptives, as well as the actual contraceptive behavior among women in Ethiopia. Intention to use contraception could be an important indicator of the potential demand for family planning services in Ethiopia	Not given	Not detailed	About 44.1% of women who were not current users of contraceptives reported that they intended to use contraceptives in the future Women who were married after the age of 20 years (OR = 1.39; 95% CI = 1.09, 1.78), who were Oromo (OR = 1.95; 95% CI = 1.49, 2.54) or other ethnic groups (OR = 1.63; 95% CI = 1.22, 2.18), who received primary (OR = 1.51; 95% CI = 1.20, 1.89) or secondary education (OR = 1.63; 95% CI = 1.05, 2.55), who were categorized as rich in the wealth index (OR = 1.27; 95% CI = 1.01, 1.62); who were employed (OR = 1.23; 95% CI = 1.02, 1.49); who were told of family planning at a health facility (OR = 1.34; 95% CI = 1.07, 1.67); whose ideal number of children was in the 3–4 range (OR = 1.47; 95% CI = 1.10, 1.97); and whose husband wanted fewer children (OR = 1.33; 95% CI = 1.09, 1.61) were more likely to have the intention to use contraceptives than their counterparts Mass media exposure, location of residence, abortion experience, and education of the husband, were significant in the analyses of contraceptives use but were not significant in the analyses of intention to use contraceptives
2016	N. van der Westhuizen and G. Hanekom SOUTH AFRICA	Cross‐sectional survey	To determine the quantity and quality of knowledge about the IUCD, and to evaluate its acceptability for future use in South Africa	Not given	Not detailed	Despite the availability of the IUCD in SA clinics and hospitals, its uptake is poor. Awareness of this method seems to have improved over the past few years, but the qualitative knowledge is still considerably lacking. Forty‐five percent (*n* = 86) of patients indicated a desire for future IUCD use
2017	Francesca L. Cavallaro, Lenka Benova, David Macleod, Adama Faye, and Caroline A. Lynch SENEGAL	Cross‐sectional survey	To analyze FP trends among harder‐to‐reach groups (including adolescents, unmarried and rural poor women) in Senegal	Coale's framework of fertility decline	DHS measures used ITU as a proxy for willingness	intention to use FP has remained stable at around 40% since 2005 for all groups except unmarried women (75% of whom intend to use) Intention to use was consistently highest among unmarried women with unmet needs, rising to 75% in 2014. In contrast, among easier‐to‐reach women, adolescents, and the rural poor, intention to use FP rose between 1997 and 2005, and has since stagnated around 40%. Among women with unmet need, the proportion with neither knowledge of nor intention to use FP was lower than the proportion with both knowledge and intention by 2014, in all groups except for adolescents (Supplementary material 2). There is no clear association between intention to use and woman's age, parity, or wealth. Intention to use also increased with the number of living children, although urban and better educated women no longer had higher odds of intention to use
2017	Nadine Shaanta Murshid PAKISTAN	Cross‐sectional survey	To examine the association between reports of IPV and the use of contraceptives in Pakistan	Not given	Not detailed	If lifetime prevalence of physical violence and emotional violence increased by one unit each, the relative risk for intending to use contraceptives increased significantly by a factor of 2.42 and 1.97, respectively. Policy and practice implications are discussed. The key finding that the experience of intimate partner violence was associated with increased use of contraceptives is in line with studies from neighboring Bangladesh. This association suggests that women experiencing intimate partner violence use contraceptives as a tool to gain control over their own bodies and their own futures
2017	Veronika V. Mesheriakova and Kathleen P. Tebb USA	Prospective cohort study	To examine the effectiveness of an iPad‐based application (app) on improving adolescent girls’ sexual health knowledge and on its ability to influence their intentions to use effective contraception among girls aged 12–18 years recruited from three school‐based health centers in California	Not given	Not detailed	After using the app, 68% of the sexually active participants reported intention to use effective contraception in the future, and sexual health knowledge improved significantly to 79% (*p* < 0.001). The most popular method was the birth control pill, with 23% of participants indicating future intention to use this method
2017	Rajesh Kumar Rai, ETHIOPIA	Cross‐sectional survey Multivariate binary logistic regression analysis	To examine whether the sex composition of living children and future desire for additional children were associated with the intention to use contraceptives among Ethiopian women aged 15–49	Social cognitive approach	Not detailed	Irrespective of the sex composition of living children and desire for additional children, future intention to use contraceptives increased significantly between 2000 and 2011 Women who had at least one child (with an equal number of boys and girls, more boys than girls or more girls than boys) who did not want any more children, and those who were unsure about their desire for additional children, showed an increased intention to use contraceptives in the future, compared with those with an equal number of boys and girls who expressed a desire for additional children. Women with no children and who did not want children, or those who were unclear about their future desire, showed a lower intention to use contraceptives, compared with women with an equal number of boys and girls who wanted a child in the future If women or couples are not satisfied with their existing (sex) composition of children, their plan to have an additional child will discourage future intention to use contraceptives, Looks at intention but not use
2017	Elena Fuell Wysong, Krystel Tossone, and Lydia Furman USA	Cross‐sectional survey	To examine whether low‐income inner‐city expectant women who intend to breastfeed make different contraceptive choices than those who intend to formula feed in expectant women age 14 years and older receiving prenatal care at MacDonald Women's Hospital the USA	Not given	Not detailed	Women intending to breastfeed had 0.44 times the odds of intending to use birth control after delivery (95% CI [0.19, 1.05], p 1⁄4 .06), while women intending to feed formula had 2.26 times the odds of intending to use birth control after delivery (95% CI [0.95, 5.40]). Contraceptive attitudes significantly impacted intent to use contraception (p 1⁄4 .007), with every point higher on the contraception attitudes scale equating to a 7% increase in odds of postpartum contraception use Postpartum contraceptive intentions do not differ significantly between women intending to breastfeed and those intending formula feeding. Contraception attitudes do not significantly change this association but were significantly related to contraceptive intent Intent to breastfeed at all or exclusively did not significantly impact odds of intending to use contraception after delivery, of intending to use a LARC method after delivery, or of intending to begin contraception postpartum prior to discharge
2017	Caroline Wuni, Cornelius A. Turpin, and Edward T. Dassah GHANA	Cross‐sectional survey	To determine factors influencing current use and future contraceptive intentions of women who were attending child welfare clinics within two years of delivery in Sunyani Municipality, Ghana	Not given	Not detailed	Family planning discussions during child welfare clinic (adjusted RR, 1.12; 95% CI, 0.99, 1.26) or with one's spouse (adjusted RR, 1.20; 95% CI, 1.08, 1.34), desire to space children (adjusted RR, 1.35; 95% CI, 1.17, 1.55), previous (adjusted RR, 1.15; 95% CI, 1.05, 1.27) and current (adjusted RR, 1.11; 95% CI, 1.01, 1.22) contraceptive use were predictive of clients’ intention to adopt family planning in the future
2018	Amy G. Bryant, Ilene S. Speizer, Jennifer C. Hodgkinson, Alison Swiatlo, Siân L. Curtis, and Krista Perreira USA	Cross‐sectional survey	To understand practices, preferences, and barriers to the use of contraception for women obtaining abortions at clinics in North Carolina.	Not given	Not detailed	Almost one‐third of women (29%) reported that they had wanted to use contraception in the last year but were unable Approximately three‐fourths of respondents (76%) stated that they intend to use contraception after this pregnancy. Approximately one‐fifth of women stated that would like to use long‐acting reversible contraception (LARC) after this abortion, with the most common method desired being the oral contraceptive pill
2018	Victoria Shelus, Lauren VanEnk, Monica Giuffrida, Stefan Jansen, Scott Connolly, Marie Mukabatsinda, etc RWANDA	Mixed method study	To explore the impact of a serial radio drama on fertility awareness and other factors in Rwanda	Not given		No significant differences in modern family planning use or intention to use were found between listeners and nonlisteners, but listeners reported greater supportive norms, self‐efficacy, and discussion of family planning
2018	Bola Lukman Solanke, Olufunmilola Olufunmilayo Banjo, Bosede Odunola Oyinloye, Soladoye Sunday Asa NIGERIA	Cross‐sectional survey Unadjusted multinomial logistic regression	To examine the association between grand multiparity and intention to use modern contraceptives in Nigeria.	The theory of planned behavior	This information was sourced from currently married women who were not using a modern method. The variable has three categories, namely, (1) use later, (2) uncertain, and (3) does not intend to use.	Results further revealed pregnancy termination, fertility planning status, exposure to mass media family planning messages, knowledge of modern contraceptives, ideal family size, remarriage, household power relations, and maternal education as other key factors influencing expected risk of intention to use contraceptives relative to being uncertain about future contraceptive use
2018	Tom Lutalo, Ron Gray, John Santelli, David Guwatudde, Heena Brahmbhatt, Sanyukta Mathur, David Serwadda, Fred Nalugoda, and Fredrick Makumbi UGANDA	Prospective cohort study	To estimate rates of an unfulfilled need for contraception, defined as having the unmet need and intent to use contraception at baseline but having an unintended pregnancy or with persistent unmet need for contraception at follow up of sexually active nonpregnant women with unmet need for contraception in Uganda	Not given	“Do you intend to use any contraceptive method between now and your next pregnancy?”	Significant discrepancy between women's intent to use contraception (text greater 60%) and subsequent initiation of use
2018	Sumitra Dhal Samanta, Gulnoza Usmanova, Anjum Shaheen, Murari Chandra, and Sunil Mehra INDIA	Cross‐sectional survey	to describe the design, implementation, and baseline findings of “family‐centric safe motherhood approach among marginalized young married couples in rural India” with the primary aim to improve the reproductive health of just married and first time pregnant couples in India	Not given	Not detailed	Almost all respondents were knowledgeable about at least one method of contraception, and this indicator is consistent in two blocks. On the other hand, slightly more recent parents discussed about family planning choices/use with their partners compared to recently married young couples. Furthermore, 64.3% of respondents have an intention to use any family planning method in the next 12 months
2018	Heba Mahmoud Taha Elweshahi, Gihan Ismail Gewaifel, Sameh Saad EL‐Din Sadek, and Omnia Galal El‐Sharkawy EGYPT	Cross‐sectional survey	To estimate the level of unmet need for postpartum family planning one year after birth as well as identify factors associated with having unmet needs in Alexandria, Egypt	Not given	Not detailed	Perceived susceptibility to conceive and intention for future use of contraceptives Less than a half of women (47.1%) perceived that they are susceptible to pregnancy at any time however 36.3% stated that they are not susceptible at all. The remaining 16.7% were not sure. On the other hand, only 59.3% of women intended to use a method in the near future, 20.1% of them do not intend to use a modern contraceptive soon and 20.6% are not sure
2018	Teklehaymanot Huluf Abraha, Hailay Siyum Belay, Getachew Mebrahtu Welay ETHIOPIA	Cross‐sectional survey	To assess intention to use modern contraceptives and to identify factors associated among postpartum women in Aksum town, Ethiopia	Not given	Not given	Intention to use modern contraceptives was 84.3%. Resumed sexual intercourse (AOR = 1.78; 95% CI: 1.34, 3.92) and women whose their husbands approved family planning to use (AOR = 1.57; 95% CI: 2.02, 5.57) were more likely to have intention on contraceptive use. In addition, those women who knew at least one method of modern contraceptive (AOR = 5.17; 95% CI: 1.69, 15.82) were more likely to have intention to use a modern contraceptive during the extended postpartum period compared to their counterparts.
2018	Zhongchen Luo, Lingling Gao, Ronald Anguzu, Juanjuan Zhao CHINA	Cross‐sectional survey	To describe the intentions of and barriers to the use of long‐acting reversible contraceptives (LARCs) in the postabortion period among women seeking abortion in mainland China	Not given	“Would you like to use an intrauterine device (IUD) for contraception in the immediate postabortion period?” “Would you like to use an implant for contraception in the immediate postabortion period?”	This study identified that only around two‐fifths of Chin‐ ese women seeking abortion were interested in using a LARC. One‐third intended to use IUDs, while only one in seven intended to use the implant method during the postabortion period Intention to use LARCs was predicted by marital status, frequency of sexual activity, number of children, planned timing of next pregnancy, and previous LARC use. Impaired future fertility, being harmful to health, irregular bleeding, risk of complications, and lack of awareness with regard to LARCs were the main barriers in their potential use.
2018	Ana Luiza Vilela Borges, Osmara Alves dos Santos, and Elizabeth Fujimori BRAZIL	Prospective cohort study	To examine the effect of pregnancy planning status in the concordance between intention to use and current use of contraceptives among postpartum women primary health care facilities in São Paulo, Brazil	Not given	To assess women's intentions to use contraceptives, we asked them while they were pregnant what type of contraceptive they intended to use after childbirth	On average, we observed 28.9% concordance between contraceptive preference and contraceptive use, which means that less than a third of postpartum women were actually using the method that they reported they intended to use after childbirth. Postpartum women whose pregnancy was unplanned were less likely to use the contraceptive methods that they intended to use when they were pregnant [aOR = 0.36; 95% CI = 0.14, 0.97; *p* = 0.044] Substantial differences between the contraceptive methods used by Brazilian postpartum women and the methods they intended to use when they were pregnant, this varied by FP method
2018	Jody R. Lori, Meagan Chuey, Michelle L. Munro‐Kramer, Henrietta Ofosu‐Darkwah, and Richard M. K. Adanu GHANA	Prospective cohort study	To examine the uptake and continuation of family planning following enrolment in group versus individual ANC in Ghana	Not given	Immediate postpartum intention to use family planning	Women who participated in group ANC were more likely to use modern and nonmodern contraception than those in individual care (59.1% vs. 19%, *p* < 0.001). This relationship improved when controlled for intention, age, religion, gravida, and education (AOR = 6.690; 95% CI: 2.724, 16.420). Women who stated immediately postpartum that they intended to use contraception had higher odds of using a family planning method (modern or nonmodern) one‐year postpartum than those with no stated intention (OR = 2.171; 95% CI: 1.109, 4.250).
2018	Sebastian Eliason, Frank Baiden, Derek Anamaale Tuoyire, and Kofi Awusabo‐Asare GHANA	Cross‐sectional survey	To better understand the issues by examining the sex composition of living children and how it is associated with reproductive outcomes such as pregnancy intendedness and intention to use postpartum family planning among women in a matrilineal area of Ghana	Not given	The intention to adopt postpartum family planning (PPFP)	Sex composition of living children had a significant association with pregnancy intendedness but not with intention to use postpartum family planning Here was persistence of more sons than daughters born in a predominantly matrilineal inheritance system and sex composition of living children had a significant association with pregnancy intendedness but not with intention to use postpartum family planning
2018	Bobby Kgosiemang and Julia Blitz BOTSWANA	Cross‐sectional survey	To assess the level of knowledge, attitudes, and practices of female students with regard to emergency contraception at the University of Botswana	Not given	Not detailed	Better knowledge of emergency contraception was associated with more positive attitudes towards actual use (*p* < 0.001). Older students (*p* < 0.001) and those in higher years of study (*p* = 0.001) were more likely to have used emergency contraception
2019	Philile Shongwe, Busisiwe Ntuli, and Sphiwe Madiba, ESWATINI	Qualitative, Focus group discussions	To explore the views of Eswatini men about the acceptability of vasectomy	Not given	Not detailed	Intention to use vasectomy was very low Cultural beliefs, societal norms, lack of knowledge about the procedure for vasectomy, and misconceptions influenced the acceptability of vasectomy greatly Ideas about vasectomy were influenced by their misconceptions not based on facts
2019	Natasha A. Johnson, Elena Fuell Wysong, Krystel Tossone, and Lydia Furman USA	Pre‐ and postsurvey	To understand how women's prenatal infant feeding and contraception intentions were related to postpartum choices in of expectant women ‡14 years of age receiving care at MacDonald Women's Hospital, Cleveland, Ohio	Not given	Not recorded in the pretest, only posttest	Prenatal feeding and contraceptive intent were significantly associated with postpartum feeding and contraceptive choice Prenatal contraceptive intention was significantly associated with a contraceptive choice both postpartum in‐hospital and at the postpartum visits (PPV) No statistically significant relationship between the three feeding choice variables (exclusive breastfeeding versus mixed feeding vs. exclusive formula feeding) and contraceptive choice Thus, mothers who were breastfeeding at all, as compared with those who were feeding formula exclusively, were less likely to have effective contraception
2019	Hailay Syum, Gizienesh Kahsay, Teklehaymanot Huluf, Berhe Beyene, Hadgu Gerensea, and Gebreamlak Gidey, and others ETHIOPIA	Cross‐sectional study	To assess intention to use LAPMs and their determinants among short‐acting users in health institutions among women who are short‐acting contraceptive users that visited health institutions Aksum Town, North Ethiopia	Not given	Desire to use long‐acting and permanent contraception methods as reported by the study participant	Good knowledge on LAPMs [AOR = 2.15; 95% CI (1.29, 3.56)], positive attitude towards LAPMs [AOR = 3.41; 95% CI (1.99, 5.85)], 18–24 years of age [AOR = 3.18; 95% CI (1.30, 7.79)], being primary school in educational level [AOR = 0.34; 95% CI (0.14, 0.78)], decision on the number of children jointly with partner [AOR = 2.05; 95% CI (1.01, 4.18)], having more than two children [AOR = 10.67; 95% CI (1.29, 88.31)], and no [AOR = 10.21; 95% CI (3.10, 33.58)] and one [AOR = 4.70; 95% CI (1.68, 13.13)] extra number of children desired were factors significantly associated with having intention to use LAPMs
2019	Fauzia Akhter Huda, John B. Casterline, Faisal Ahmmed, Kazuyo Machiyama, Hassan Rushekh Mahmood, Anisuddin Ahmed, and John Cleland BANGLADESH	Cross‐sectional survey	To understand how Bangladeshi women's perceptions of a method's attributes are associated with their intention to use that method. To examine associations between 12 method attributes and intention to use the pill or the injectable in Bangladesh	Not given	Intended to do so in the next 12 months and whether they intended to do so at any time in the future.	The likelihood that a woman intended to use a method was positively associated with her perception that it is easy to use (odds ratio, 2.9) and does not cause serious health problems (1.7) or affect long‐term fertility (2.9). Satisfied past users of a method were more likely than never users to report intending to use the method (5.2). Intention to use the pill rather than the injectable was positively associated with education (2.0–3.6) and having a migrant husband (1.7). In the adjusted model, however, only four method attributes remained associated with intention to use the pill or injectable. Women had elevated odds of intending to use a method if they perceived it to be easy to use (odds ratio, 2.9), did not cause any serious health problems (1.7), and did not affect long‐term fertility (2.9). In addition, satisfied past users were more likely than never users to report intending to use the pill or injectable (5.2).
2019	Aalaa Jawad, Issrah Jawad, and Nisreen A. Alwan GLOBAL	Systematic review	To evaluate the effectiveness of interventions using social networking sites (SNSs) to promote the uptake of and adherence to contraception in reproductive‐age women	Not given	Not detailed	They found no differences in the use of condoms at the last act of sexual intercourse at six months or the intention to use condoms between the intervention and control groups
2019	Sara Adelman, Caroline Free, and Chris Smith CAMBODIA	Randomized controlled trial	To evaluate which characteristics collected at the point of abortion are associated with contraceptive use over the extended postabortion period for women in Cambodia	Not given	Not detailed	Prior use of contraception, intention to use postabortion contraception, increased number of children, and medical abortion were associated with increased contraceptive use over the year postabortion. Contraceptive intention was the strongest predictor of continuation in the four‐month period postabortion. More than half the women in the study were undecided on their intention
2019	Ona L. McCarthy, Hanadi Zghayyer, Amina Stavridis, Samia Adada, Irrfan Ahamed, Baptiste Leurent, Phil Edwards, Melissa Palmer, and Caroline Free PALESTINE	Randomized controlled trial	To estimate the effect of a contraceptive behavioral intervention delivered by mobile phone text message on young Palestinian women's attitudes towards effective contraception among women aged 18–24 years living in the West Bank, who were not using contraception.	Not given	Not detailed	Intervention control was more likely to agree that their friends would use an effective method and to intend to use an effective method, compared to participants in the control group The intervention moderately improves knowledge about effective contraception, perceived norms about friends using effective contraception, and intention to use effective contraception.
2019	Silas Ochejele,; Chris Ega, Muhammad Shakir Balogun, Patrick Nguku, Tunde Adedokun, and Hadiza Usman NIGERIA	Cross‐sectional study	To determine the factors that affect the contraceptive intentions of women who survive SAMM in Kaduna State, northern Nigeria.	Not given	Not detailed	Only 56 out of the 69 respondents who reported not wishing to have another child in the future responded to this question. The commonest reason given for not wanting to get pregnant again was to avoid complications (17 women [30.4%]) The majority of the respondents (228 women [69.1%]) intended to use contraception in the future. Having more than five living children and birth interval were found to be significant predictors of intention to use modern contraception in SAMM survivors, the same predictors as for FP use
2019	Crystal L. Moore, Alison H. Edie, Jennifer L. Johnson, and Eleanor L. Stevenson USA	Pre‐ and postintervention survey	The LARC QI intervention Project aims were threefold: (1) increase knowledge about LARC by 50% among college students attending group educational sessions one month after implementation; (2) increase intention to use a LARC method by 20% among college students one month after the implementation of the LARC‐QI project, and (3) increase usage of LARC methods three months after implementation of the project in the USA	Not given	Not detailed	Intention to use also increased compared to reported actual use.
2019	E. Costenbader, S. Zissette, A. Martinez, K. LeMasters, and N. A. Dagadu (Costenbader, et al. 2019) DRC	Cross‐sectional survey	18–22 college students in DRC	Theory of planned behavior	On the survey, respondents were asked how likely they were to use modern contraceptives in the future. This question was asked on a four‐point Likert scale, with one being extremely unlikely to use and four being extremely likely to use modern contraceptives in the future	Social norms influence intentions to use modern contraception overall, but that normative influence varies by gender In both the men's and women's models, multiple factors were associated with intention to use family planning, there was not one common factor that was significantly associated with the outcome for both men and women. Common to both models was the fact that respondent age, having transportation to an FP clinic, relationship quality, and couple communication were not significantly associated with intention to use a modern method. In addition, none of the social norm constructs had significant indirect effects (i.e., via relationship quality or couple communication) on intention to use a modern method.
						Among the women, the variables that were significantly associated with intention to use a modern contraceptive method were the number of children the respondent had, the information she had about FP, and perceptions of two social norms; injunctive FP norms and descriptive gender equity norms related to childcare responsibilities. Among the men, having the means to purchase contraception, an understanding of the availability of FP methods in the community, and perceptions of descriptive FP social norms were significantly associated with intention to use a modern method of contraception Specifically, we found that the descriptive norm around FP use was only influential for men and that the injunctive norm related to FP use was only influential among the women. These results indicate that men are more swayed by their perceptions about how many of their peers are using FP, whereas women are more concerned about social sanctions resulting from the use of FP
2019	Andrea L. DeMaria, Beth Sundstrom, Amy A. Faria, Grace Moxley Saxon, and Jaziel Ramos‐Ortiz USA	Cross‐sectional survey	To assess combined oral contraceptive pill (COC) users’ intention to obtain LARC	Theory of planned behavior (TPB)	TPB factors were measured and adapted from Ajzen's Likert scales	Self‐identity and attitude pathways were significant (*p* < 0.01) toward intention Participants’ attitudes, behaviors, perceived behavioral control, and self‐identity influence LARC intention Self‐identity with being a pill user had a significant, negative relationship with LARC intention Current COC user's intention to switch to a LARC method in the prolonged future proved greater than intention for immediate use (12 months)
2019	Dana Sarnak, Amy Tsui, Fredrick Makumbi, Simon Kibira, and Saifuddin Ahmed UGANDA	Cohort study	To assess the dynamic influence of unmet need on time to contraceptive adoption, as compared with that of contraceptive intentions and their concordance in Uganda	Not given	Whether they would use contraceptives in the future, and their responses were classified as (1) intend to use and (2) do not intend to use	The unmet need indicator underperforms in predicting future contraceptive adoption compared to contraceptive intentions, which merits further consideration as a complementary predictor of future use. Non contracepting women with unmet needs but no intention to use contraception in particular warrant programmatic attention The study found women classified as having unmet needs were slower to adopt contraception than those without unmet need, after adjustment for background covariates (aHR = 0.79, 95% CI = 0.57, 1.10). Women intending future contraceptive use were significantly faster to adopt (aHR = 1.45, 95% CI = 1.22, 1.73) than those not intending. Women with no unmet need but intending to use had the highest rate of adoption compared to those with no need and no intention to use (aHR = 2.78, 95% CI = 1.48, 5.25). His study assesses the dynamic influence of unmet need on time to contraceptive adoption, as compared with that of contraceptive intentions and their concordance
2020	Janine Barden‐O'Fallon, Jennifer Mason, Emmanuel Tluway, Gideon Kwesigabo, and Egidius Kamanyi TANZANIA	Pre post intervention study (no control)	To assess the effect of providing revised injectable and HIV risk counseling messages on contraceptive knowledge and behavior during a three‐month pilot intervention in Tanzania.	Not given	Not detailed	Results indicate that the counseling messages did not cause a decrease in the uptake of injectables (Depo‐Provera): 97% of interviewed clients received Depo‐Provera at their visit; 60% reported an intention to use condoms for dual protection

From 1990 to 1999, seven papers on ITU were published. This increased to 28 papers from 2000 to 2009, of which over half related to condom use. From 2010 to 2020, 76 papers on ITU were published, and more than 50 papers came out in between 2015 and 2020. Sixty‐eight papers used cross‐sectional study designs and 15 longitudinal studies using cohorts to look at the relationships between variables over time. There were 15 experimental and quasi‐experimental studies, seven of which were randomized‐control trials (RCTs). There were three mixed‐method studies, three qualitative studies, three reviews, and two studies of psychometric scales.

The surveys that we included contain the Demographic Health Surveys (DHS), the Measure Evaluation Family Planning, and Reproductive Health Indicators Database, CDC RH assessment toolkit in a conflict setting, PMA2020, Track20/FP2020, Multiple Indicator Cluster Surveys, Reproductive Health Surveys (CDC), Breakthrough Action Research Indicators, National Survey of Family Growth (US), and NATSAL (UK).

### Synthesis of Results

#### What Theoretical Frameworks Are Used?

Of the 112 papers include, 67 papers did not state or explain how they understood intention worked. Twenty‐five papers used Azjen's (1991) theory of planned action or a variation of it, in which attitudes, subjective norms, and perceived behavioral control, influence an individual's intentions towards a given behavior; such intentions predict whether an individual engages in that behavior in the future (Nguyen, Saucier, and Pica 1996; Gagnon and Goden 2000; Fazekas, Senn, and Ledgerwood 2001; Wang et al. 2004; Rosengard et al. 2005; O'Connor, Ferguson, and O'Connor 2005; Cha, Kim, and Doswell 2007; Wang et al. 2008; Kang et al. 2008; Heeren et al. 2009; Raine et al. 2011; Brown, Hurst, and Arden 2011; Lee et al. 2011; Potard et al. 2012; Picavet, Vlugt, and Wijsen 2014; Raine‐Bennett and Rocca 2015; Kim et al. 2015; Lee, Carvallo, and Lee 2015; Hanson et al. 2015; Roderique‐Davies et al. 2016; Solanke, Oyinloye, and Asa 2018; Costenbader et al. 2019; DeMaria et al. 2019). Twelve papers referred to other behavioral theories of intention including the health belief model (three papers), diffusion theory (three papers), and ideation theory (two papers), or specific constructs like self‐efficacy (four papers). Seven of the papers used more bespoke models that were not easily categorized under established behavioral models, such as the social‐psychological perspectives (Zimmers et al. 1999), prospect theory (O'Connor, Ferguson, and O'Connor 2005), synthesis framework (Agha 2010); the extended parallel process model (EPPM) (Campo et al. 2012); social ecological theory (Irani, Speizer, and Fotso 2014); and the information motivation and behavioral framework (Onono et al. 2014) and integrated behavioral model (Williams et al. 2008).

### How ITU Is Operationalized

Across the papers, there is no accepted definition or measure of intent to use contraception. Over 50 papers did not state the exact wording of the items and, of those that did, few of the measures were validated. There are two notable exceptions. Raine‐Barrett and Rocca 2015 (2015) developed the Contraceptive Intent Questionnaire (CIQ) that measures contraceptive intent among adolescent girls in the USA, based on a theory of reasoned action and necessity‐concerns framework. The CIQ has modest reliability and good internal validity. Lee, Carvallo, and Lee (2015) developed another measure for determining the degree to which Korean immigrant women in the USA intend to use oral contraceptives that was both valid and reliable.

Of the papers that did specify the exact wording used to measure ITU, twenty‐four used the items from the Demographic and Health Survey (DHS) or a slight variation of it. The way that the DHS measures are applied in these papers is not standard or consistent. We found that 10 papers used the question (or a variation of it), Do you think you will use a contraceptive method to delay or avoid pregnancy at any time in the future?, six papers used the question, Do you intend to use a method within the next 12 months, and eight papers used a combination of the two questions. Another 15 papers defined ITU in relation to a specific method (e.g., emergency contraceptive pill, condoms, and long acting and permanent methods (APM) or to a specific time frame (e.g., postdelivery or abortion),

Twenty papers drew on theory of planned/reasoned behavior to inform their items (Gagnon and Godin 2000; Fazekas, Senn, and Ledgerwood 2001; Wang et al. 2004, 2008; Rosengard et al. 2005, Williams et al. 2008; Kang and Moneyham et al. 2008; Raine et al. 2011; Raine‐Barrett and Rocca 2015; Brown, Hurst, and Arden 2011; Lee et al. 2015, 2011; Potard et al. 2012; Picavet, Vlugt, and Wijsen 2014; Kim et al. 2015; Hanson et al. 2015; Roderqiue‐Davies 2016; Costenbader et al. 2019; DeMaria et al. 2019). Five papers used bespoke theories to inform their measures (Campo et al. 2012; Irani, Speizer, and Fotso 2014; Onono et al. 2014; Agha 2010; Larsson et al. 2004). Of these items that were explicitly informed by theory, one‐third (seven papers) aligned with the DHS items.

### How ITU Is Used

The review found that ITU has become increasingly popular over time. Of the 112 papers found in this scoping review, 92 papers treated ITU as a dependent variable (alone or in some combination with knowledge and attitudes, and contraceptive use). There are three broad ways that ITU has been used in the evidence found.

The first way ITU has been applied was to augment the current measures of unmet need. Several authors have incorporated information on women's future contraceptive intentions to distinguish contraceptive readiness of women with unmet needs (Moreau et al. 2019; Ross and Winfrey 2001; Roy et al. 2003; Cavallaro et al. 2017; Sarnak et al. 2020; Khan 2015; Callahan and Becker 2014; Moreau et al. 2019).

The second way ITU was used was to measure ideational formation and outcome for those developing and implementing behavior change programming. Twelve papers used ITU, as opposed to self‐reported contraceptive use, as the outcome measure for counseling and social and behaviour change communication (SBCC) interventions and programs. (Shelus et al. 2018; Amin and Sato 2004; Zimmers et al. 1999; Krenn, et al. 2014; Jawad, Jawad, and Alwan 2019; McCarthy et al. 2019; Moore et al. 2019; Graham et al. 2002; Agha and Van Rossem 2002; Brown, Hurst, and Arden 2011; Onono et al. 2014; Barden‐O'Fallon et al. 2020).

The final way ITU was used in the papers was to predict future use of contraception. Fifteen papers assessed the relationship and causal pathways between ITU and contraceptive use (Curtis and Westoff 1996; Kvalem et al. 2000; Roy et al. 2003; Ross and Winfrey 2001; Callahan and Becker 2014; Lori et al. 2018; Tang 2016; Johnson et al. 2019; Adelman, Free, and Smith 2019; Raine et al. 2011; Lutalo et al. 2018; Haile et al. 2016; Akelo et al. 2013; Potard et al. 2012; French, Albanese, and Gossett 2016). ITU was also used to identify the determinants that predict contraception use.

### Reported Results

Where ITU augmented the current measures of unmet need, Sarnak et al. (2020) found that women classified as having unmet need were slower to adopt contraception than those without unmet need in Uganda; however, women intending future contraceptive use were significantly faster to adopt than those not intending. Women with no unmet need but intending to use had the highest rate of adoption. Two papers indicate that women move in and out of the unmet need grouping depending on a range of variables (Callahan and Becker 2014; Ross and Winfrey 2001).

Where ITU was used was to measure ideational change, 14 papers used ITU as an outcome measure to assess Social and Behavior Change programs (such as radio and TV shows, phone, iPad, mass and social media, and school and community‐based education). Two papers looked at specific counseling on long‐acting reversible contraceptives (LARCs) and found that counseling was associated with increased ITU (Barden‐O'Fallen et al. 2020; Moore et al. 2019). The fact that only two papers looked at ITU in the context of counseling interventions reinforces an observation made by Raine et al. (2011): there has been little attention on how to support women with low intentions in clinical settings.^4^

Where papers used ITU to assess the relationship and causal pathways between ITU and use of contraceptives, five prospective cohort studies have found that intention to practice contraception is a strong predictor of subsequent use in Bangladesh, Ghana, India, Morocco, and the USA (Curtis and Westoff 1996; Roy et al. 2003; Callahan and Becker 2014; Lori et al. 2018; Tang et al. 2016; Johnson et al. 2019). Ross and Winfrey's (2001) secondary analysis of DHS data in 27 countries found that for each increase of one percentage point in stated ITU contraceptives, there was nearly a 1% rise in the actual use of contraceptives. ITU also has been found to have a positive relation to contraceptive continuation; Raine et al. (2011) and Adelman, Free, and Smith (2019) found that a higher intent to use a method was associated with a lower risk of discontinuation. However, five papers found null or negative associations between ITU and use (Lutalo et al. 2018; Hale et al. 2016; Akelo et al. 2013; Potard et al. 2012; French, Albanese, and Gossett 2016). In an RCT in Norway, Kvalem et al. (2000) found a relationship between adolescents’ actual use of condoms at the most recent occasion of sexual intercourse and their intention to do so the next time (Kvalen et al. 2000).

The majority of studies looked at ITU in cross‐sectional studies at national, facility, and household levels. The cross‐sectional studies explored the variables that were associated with ITU contraception and suggest that ITU contraception is affected by many personal and social variables. These can be grouped into broader categories: (1) socioeconomic variables such as social and economic factors that indicate a person's status within a community; and (2) behavioral variables, for example, an individual's knowledge and attitude (see Table A2). The socioeconomic variables had a positive relationship with ITU and included higher educational attainment, number of children, and partner's support for family planning. The behavioral variables that had a positive relationship with ITU included a positive attitude to contraception, support from those immediately around the person (such as partners and friends), knowledge about contraceptive methods, and previous experience using contraception. These studies, however, provide little information about the relations between the variables over time or the causal relationship between them.

Several studies focused on ITU for a specific method: condoms (15 papers), LARC methods (14 papers), and emergency contraceptive pills (ECP; six studies). These studies suggest that intentions may vary by the contraceptive method under consideration. For example, anxieties about longer‐term effects may be relevant to LARCs but not to condoms. The assessment by Kim et al. (2015) of ITU multiple methods in Korea found possible influences of ECP awareness on ITU other contraceptives.

Another finding is that there can be degrees of intentions. Several studies attempted to decipher the strength of intention (Solanke, Oyinloye, and Asa 2018; Kim 2015). Using DHS data in Nigeria, Solanke, Oyinloye, and Asa (2018) found that certain background characteristics had degrees of association with ITU. Rural residence was associated with higher ITU and advanced reproductive age and religion (compared to no religion) were associated with lower ITU. This suggests that it is possible to distinguish degrees of intention that could be relevant to programming.

## DISCUSSION

In this scoping review, we identified 112 peer‐reviewed journal articles, assessed nine surveys, and scoped the theoretical literature on intention from behavioral science. Our findings indicate that measures of ITU contraception are being used in a variety of ways and in much research, suggesting the importance and utility of this construct. It may be particularly relevant in assessing programs focused on ideational change and it may help distinguish between women within the category of unmet need. By synthesizing the evidence, five prospective cohort studies have found that intention to practice contraception is a strong predictor of subsequent use and suggests it has the potential to predict contraceptive uptake and use in its own right (Curtis and Westoff 1996; Roy et al. 2003; Callahan and Becker 2014; Lori et al. 2018; Tang et al. 2016; Johnson et al. 2019). However, its definition and operationalization have been underelaborated and inconsistent.

What is notable is that the current operationalization of ITU is driven by the relevant items in the DHS and disconnected from advances in behavioral science. In behavioral science, intentions signal the end of a person's deliberations about what actions one will perform, how hard one will work for it, and how much effort one will apply to achieve the desired outcomes (Ajzen 1991; Gollwitzer 1993; Webb and Sheeran 2006; Gollwitzer and Sheeran 2008). Sheeran (2002), among others, argues that moving from intention to action is complex and moderated by the type of desired behavior, the intention type, properties of intention, and cognitive and personality variables. Sheeran illustrates this complex relationship by grouping people into those with positive intentions who subsequently act (inclined actors), those who fail to act (inclined abstainers), those with negative intentions who do act (disinclined actors), and those with negative intentions who do not act (disinclined abstainers).

Different variables have been associated with the strength of intention and whether an intention will be realized. Take, for example, whether intentions are stable over time or whether the behavior predicted is a single action (Webb and Sheeran 2006; Sheeran 2002). A person's control over the behavior (e.g., they do not have the necessary knowledge, resources, opportunity, availability, or cooperation) and the potential for adverse social reaction can limit the realization of an intention (Sheeran 2002; Webb and Sheeran 2006). Moreover, intentions do not always successfully translate into behavior because committing to achieve a behavior does not necessarily prepare people to deal with the issues faced when trying to achieve it. Gollwitzer and Sheeran (2008) helpfully distinguish between a goal intention and an implementation intention. Goal intentions relate to what people plan to do some time in the future, whereas implementation intention is more specific in terms of when, where, and how one intends to achieve it. Implementation intentions tend to be a single action, whereas goal intentions tend to be the outcomes that can be achieved by performing a variety of single actions.

Distinguishing the type of intention is critical because implementation intentions are more likely to translate into behavior than goal intentions (Cohen 1992).^5^ Gollwitzer and Sheeran (2008) argue that goal intentions do not prepare people for dealing with the obstacles they face in initiating, maintaining, disengaging from, or overextending oneself in realizing their intentions. Whereas an implementation intention sets out the when, where, and how in advance, this kind of planning bridges the intention to behavior gap and increases the likelihood of intentions being realized. Take, for example, Joffe and Radius (1993), who see intention for condom use as several single actions linked with specific implementation intentions: to purchase or request condoms from a drug store or clinic; to convince their partner to use them and to use condoms correctly during intercourse. Calculated together, these assess intent to use condoms. Here, the intention is broken down into a set of single actions that specify the situation for performing an intended action.

These developments in behavioral theory are relevant to how we understand intention in contraceptive decision‐making and our findings of the mixed evidence about the causal relationship between intent to use and contraceptive use. A closer reading reveals that the studies that found a positive relationship between intention and use and those that found no relationships or negative relationships can be grouped by the types of intention they measured. The papers that found a positive relationship measured “implementation intention” questions, which specified a time frame and/or a specific method. The papers that did not find a relationship between ITU tended to capture goal intention through questions about intentions for contraceptive use in the unspecified future. These insights from behavioral theory merit further examination about the predictive ability of ITU using implementation intention or goal intention, as well as investigating other variables surrounding intention. Furthermore, more research is needed to examine if the degrees of intention related to socio‐demographic variations related to different types of intention, such as goal and implementation.

Behavioral theory has also shown that intention is a debated, layered, and phased construct. So it is surprising that most papers in this review did not specify how they understood intention to work and how it related to behavior. This conspicuous absence suggests that there are some implicit assumptions about the ITU construct in the family planning field. The most immediate assumption here is that there is already an accepted definition and measurement of ITU. This review clearly shows that this is not the case, even among the studies that used the more common DHS measures of ITU. This assumed definition of ITU is not informed by behavioral sciences, rather it is driven by legacies from theories of demographic transitions.

In demography, there is an assumed association between reproductive intentions toward smaller families and fertility decline, whether it is caused by changing material conditions or diffusion of ideas (Johnson‐Hanks 2008). We can trace the assumptions about ITU in the DHS measures back to Coale's (1973) “ready, willing and able” model that has become popular in describing the adoption of new forms of behavior and the subsequent generalization of these behaviors. Lesthaeghe and Vanderhoeft (2001) refined Coale's model so that the transitions were understood as a process of diffusion of ideas and technologies. The model argues that three intersecting preconditions are necessary for adopting new behavior: individual readiness, willingness, and ability. Here, the intention is linked with “readiness,” an individualistic rational‐cost benefit analysis of whether or not to have a child, and whether the benefits of preventing pregnancy compensate for the costs of using family planning. Thus utility of new behavior should be self‐evident, and the advantages must outweigh the disadvantages (Coale 1973; Lesthaeghe and Vanderhoeft 2001; Dereudde et al. 2016; Mannan and Beaujot 2006).

When designing, implementing, and assessing contraceptive services, there is an implicit assumption that contraceptive use is linked to whether a woman intends to get pregnant in the near future. This logic is reflected in the decision to move the ITU questions from the section on contraception to the section on fertility intention in Phase 3 (1992–1997) of the DHS. The DHS measures of ITU are the most commonly used; but, these have changed over time (see Table A4). The DHS ITU questions were initially located in the contraceptive section and covered three questions: Do you intend to use a method to avoid pregnancy at any time in the future? Which method would you use? and Do you intend to use (preferred method) in the next 12 months? In Phase 2 (1992–1997), the questions were moved into the fertility preferences section. In Phases 4 and 5, the question, Do you intend to use (preferred method) in the next 12 months? was dropped. In the final three rounds, the question related to Which method would you use? was dropped, and ITU was reduced to a single question, Do you think you will use a contraceptive method to delay or avoid pregnancy at any time in the future? This link between pregnancy intentions and contraceptive use may explain why ITU has received relatively less attention.

However, the relationship between reproductive intention, ITU, and contraceptive use is complicated at best (Higgins et al. 2012; Mumford et al. 2016; Aiken et al. 2016; Barrett and Wellings 2002; Moreau et al. 2013). Fertility intentions are often conflated with contraceptive motivations even though the two often do not align (Moreau et al. 2019; Cleland, Harbison, and Shah 2014). Raine‐Barrett and Rocca (2015) point out that contraceptive intention relates to ideas about preventative medication, and, therefore, its measurement must take into account a woman's specific beliefs about medicine and about contraception itself, along with beliefs about pregnancy, and not the cost‐benefit calculus of having a child (or not). There has been limited research examining the meaning and measurement of this construct despite its widespread inclusion in many major surveys.

Our scoping review had some limitations. To make our review more feasible, we searched only two databases. Given our limited data sources, we chose to include databases that covered both public health (e.g., PubMed) and the social sciences (e.g., Web of Science). To further the feasibility of this review, we limited the search period from 1990 to November 2020. From this review, we have found the earlier antecedents for ITU and in a subsequent systematic review of the relationship between ITU and contraceptive use, we have used a longer search period.

## CONCLUSION

ITU contraception is an important construct that has not been applied consistently. Despite its widespread inclusion in major surveys, there is limited research examining the meaning and measurement of this construct and this research does not reflect advances in behavioral theory and research. As an accepted measure that captures a more person‐centered measure of demand, there is much to be gained if we were more intentional in our use of intent to use. Further work is needed to develop and test measures that capture the complexity of intention, examine how intention differently relates to longer‐range goals compared to more immediate implementation, and demonstrate a positive relationship between ITU and contraceptive use.

This publication is based on research by Victoria Boydell. The findings and conclusions contained within are those of the authors and do not necessarily reflect positions or policies of the Bill & Melinda Gates Foundation.

REFERENCES

Aiken, Abigail R.
, 
Sonya
Borrero
, 
Lisa S.
Callegari
, and 
Christine
Dehlendorf
. 2016. “Rethinking the Pregnancy Planning Paradigm: Unintended Conceptions or Unrepresentative Concepts?” Perspective in Sexual and Reproductive Health
48(3): 147–151. 10.1363/48e10316
PMC502828527513444

Ajzen, Icek.

1991. “The Theory of Planned Behaviour.” Organizational Behaviour and Human Decision Processes
50: 179–211.

Alkema, Leotine.
, 
Vladimira
Kantorova
, 
Claire
Menozzi
, and 
Ann
Biddlecom
. 2013. “National, Regional, and Global Rates and Trends in Contraceptive Prevalence and Unmet Need for Family Planning between 1990 and 2015: A Systematic and Comprehensive Analysis.” The Lancet
381: 1642.
10.1016/S0140-6736(12)62204-123489750

Arksey, Hilary
, and 
Lisa
O'Malley
. 2005. “Scoping Studies: Towards a Methodological Framework,” International Journal of Social Research Methodology, 8(1): 19–32.

Barrett, Geraldine
, 
S. C.
Smith
, and 
Kaye
Wellings
. 2004. “Conceptualisation, Development and Evaluation of a Measure of Unplanned Pregnancy,” Journal of Epidemiology and Community Health
58: 426–433.1508274510.1136/jech.2003.014787PMC1732751

Barrett, Geraldine
, and 
Kaye
Wellings
. 2002. “What Is a “Planned” Pregnancy? Empirical Data from a British Study.” Social Science and Medicine
55: 545–557.1218846210.1016/s0277-9536(01)00187-3

Bradley, Sarah E. K.
, 
Trevor N.
Croft
, 
Joy D.
Fishel
, and 
Charles F.
Westoff
. 2012. Revising Unmet Need for Family Planning. DHS Analytical Studies No. 25. Calverton, MD: ICF International.

Bradley, Sarah E. K.
, and 
John B.
Casterline
. 2014 “Understanding Unmet Need: History, Theory, and Measurement.” Studies in Family Planning
45(2): 123–150. 10.1111/j.1728-4465.2014.00381.x
24931072PMC4369378

Cleland, John
, 
Sarah
Harbison
, and 
Iqbal H.
Shah
. 2014. “Unmet Need for Contraception: Issues and Challenges.” Studies in Family Planning
45: 105–122.2493107110.1111/j.1728-4465.2014.00380.x

Coale, Ansley.

1973. “The Demographic Transition Reconsidered.” In International Population Conference: Liege. 1: 53–72. Liege: IUSSP.

Cohen, J.

1992. A Power Primer.” Psychological Bulletin
112: 155–159.1956568310.1037//0033-2909.112.1.155

Colquhoun, Heather L.
, 
Danielle
Levac
, 
Kelly K.
O'Brien
, 
Sharon
Straus
, 
Andrea C.
Tricco
, 
Laure
Perrier
, 
Monika
Kastner
, and 
David
Moher
. 2014. “Scoping Reviews: Time for Clarity in Definition, Methods, and Reporting.” Journal of Clinical Epidemiology
67: 1291e1294.2503419810.1016/j.jclinepi.2014.03.013

Darroch, Jacqueline E.
, and 
Suneela
Singh
. 2013. “Trends in Contraceptive Need and Use in Developing Countries in 2003, 2008, and 2012: An Analysis of National Surveys.” The Lancet
381: 1756–62.10.1016/S0140-6736(13)60597-823683642

Dasgupta
Aisha
, 
Michelle
Weinberger
, 
Ben
Bellows
, and 
Win
Brown
. 2017. “New Users” Are Confusing Our Counting: Reaching Consensus on How to Measure “Additional Users” of Family Planning.” Global Health: Science and Practice
5(1): 6–14. 10.9745/GHSP-D-16-00328
28351876PMC5478230

Dereuddre, Rozemarjin.
, 
Bart
Van de
Putte
, and 
Piet
Bracke
. 2016. “Ready, Willing, and Able: Contraceptive Use Patterns across Europe.” European Journal of Population. 32(4): 543–573. 10.1007/s10680-016-9378-0.30976222PMC6241009

Gollwitzer, Peter M.
, and 
Paschal
Sheeran
. 2008. “Implementation Intentions and Goal Achievement: A Meta‐Analysis of Effects and Processes.” Advances in Experimental Social Psychology
38: 69–119.

Gollwitzer, Peter M
. 1993. “Goal Achievement: The Role of Intentions,” European Review of Social Psychology
4: 141–185.

Gollwitzer, Peter M
. 1999. “Implementation Intentions: Strong Effects of Simple Plans.” American Psychologist
54: 493–450.

Higgins, Jenny A.
, 
Ronna A.
Popkin
, and 
John S.
Santelli
. 2012. “Pregnancy Ambivalence and Contraceptive Use among Young Adults in the United States.” Perspectives on Sexual and Reproductive Health
44(4): 236–243. 10.1363/4423612
23231331PMC3730441

Johnson‐Hanks, Jennifer.

2008. “Demographic Transition and Modernity.” Annual Reviews in Anthropology
37: 31–55


Jones, Rachel K.
, 
Athena
Tapales
, 
Laura D.
Lindberg
, and 
Jennifer
Frost
. 2015. “Using Longitudinal Data to Understand Changes in Consistent Contraceptive Use.” Perspectives on Sexual and Reproductive Health
47(3): 131–139.2628796510.1363/47e4615PMC4976085

Lesthaeghe, Ron
, and 
Camille
Vanderhoeft
. 2001. “Ready, Willing and Able: A Conceptualization of Transitions to New Behavioral Forms,” In Diffusion Processes and Fertility Transition: Selected Processes, edited by 

J. B.
Casterline

, 240–264. Washington, DC: National Academy Press.25057587

Levac, Danielle
, 
Heather
Colquhoun
, and 
K. K.
Kelly


K.
O'Brien
. 2010. “Scoping Studies: Advancing the Methodology.” Implementation Science
5(1): 69. 10.1186/1748-5908-5-69.20854677PMC2954944

Mannan, Haidder R.
, and 
Roderic
Beaujot
. 2006. “Readiness, Willingness and Ability to Use Contraception in Bangladesh.” Asia‐Pacific Population Journal
21(1): 45–64.
Measure Evaluation
. 2020. Unmet need for family planning. https://www.measureevaluation.org/prh/rh_indicators/family‐planning/fp/unmet‐need‐for‐family‐planning. Access January 2021.

Moreau, C.
, 
Hall, K.
, 
Trussell, J.
, and 
J.
Barber
. 2013. “Effect of Prospectively Measured Pregnancy Intentions on the Consistency of Contraceptive Use among Young Women in Michigan.” Human Reproduction
28(3): 642–650.2324183810.1093/humrep/des421PMC3619965

Moreau
C
, 
Shankar
M
, and 
Helleringer
S
, 2019. “Measuring Unmet Need for Contraception as a Point Prevalence.” BMJ Global Health
4: e001581.10.1136/bmjgh-2019-001581PMC673057531543991

Mumford, Sunni L.
, 
Katherine
J Sapra
, 
Rosalind B.
King
, 
Jean F.
Louis
, and 
Germaine M. Buck
Louis
. 2016. “PREGNANCY Intentions—A Complex Construct and Call for New Measures.” Fertility and Sterility. 106(6): 1453–1462. 10.1016/j.fertnstert.2016.07.1067.27490044PMC5159192

Peterson, Herbert B.
, 
Gary L.
Darmstadt
, and 
John
Bongaarts
. 2013. “Meeting the Unmet Need for Family Planning: Now Is the Time.” Lancet
381: 1696–9.2368362010.1016/S0140-6736(13)60999-X

Sheeran, Paschal
. 2002. “Intention—Behavior Relations: A Conceptual and Empirical Review.” European Review of Social Psychology
12(1): 1–36. 10.1080/14792772143000003


Triandis, H.C.

1980. “Values, Attitudes, and Interpersonal Behavior.” Nebraska Symposium on Motivation
27: 195–259.7242748

Webb, Thomas L.
, and 
Paschal.
Sheeran
. 2006. “Does Changing Behavioral Intentions Engender Behavior Change? A Meta‐Analysis of the Experimental Evidence.” Psychological Bulletin
132(2): 249–268.1653664310.1037/0033-2909.132.2.249

Westoff, Charles F.
, and 
Luis H.
Ochoa
. 1991. “Unmet Need and the Demand for Family Planning.Institute for Resource Development” DHS Comparative Studies No. 5. Columbia, MD: Institute for Resource Development.

Westoff, Charles F
. 1978. “The Unmet need for Birth Control in Five Asian Countries.” Family Planning Perspectives. 10(3): 173–118.658326

  

**TABLE A1:** Intention to use measures in the survey instruments reviewed

Survey Instrument (year)	Intention to Use Measure
CDC RH assessment toolkit in a conflict setting^a^ (2011)	Do you think you will use a method to delay or avoid pregnancy in the next 12 months?
PMA2020 (2020)^b^	Female Questionnaire (Panel) Do you think you will use a contraceptive method to delay or avoid getting pregnant at any time in the future? When do you think you will start using a method? (Months/year) What method do you think you will use? Female Questionnaire (Cross‐Sectional) Do you think you will use a contraceptive method to delay or avoid getting pregnant at any time in the future? When do you think you will start using a method? (Months/year) What method do you think you will use?
UNICEF Multiple Indicator Cluster Surveys^c^	Not included
CDC Reproductive Health Surveys (2018)^d^	Intend to use a method in the future What method do you think you will use?
National Survey of Family Growth (US)^e^	Not included
NATSAL (UK)^f^	Not included

^a^ https://www.cdc.gov/reproductivehealth/global/tools/crisissituations.htm.

^b^ https://www.pmadata.org/data/survey‐methodology.

^c^ https://mics.unicef.org.

^d^ http://ghdx.healthdata.org.

^e^ https://www.cdc.gov/nchs/data/nsfg/NSFG‐2017‐2019‐FemaleCAPIlite‐forPUF‐508.pdf.

^f^ https://www.natsal.ac.uk/natsal‐survey/natsal‐3.

**TABLE A2:** Overview of variables’ relationship to ITU found in the studies

	Positive association with ITU	Negative association with ITU
Socioeconomic factors		
Education	Baumgarter et al. 2012; Cavallaro et al. 2017; Kuang, Ross, and Madsen 2014; Adegbola and Okunowo 2009; Syum et al. 2019; Huda et al. 2019; Mekonnen et al. 2014; Tiruneh et al. 2016; Spence, Elgen, and Harwell 2003; Di Giacomo et al. 2013; Meskele et al. 2014; Kgosiemang and Blitz 2018; Udomboso et al. 2015	
Number of children	Baumgarter et al. 2012; Cavallaro et al. 2017; Syum et al. 2019; Hamid, Stephenson, and Rubenson 2011; Luo et al. 2018; Adelman, Free, and Smith 2019; Ochejele et al. 2019; Babalola et al. 2015; Peterson 1999; Costenbader et al. 2019	Solanke, Oyinloye, and Asa 2018; Udomboso et al. 2015
Abortion	Adelman, Free, and Smith 2019; Mogilevkina and Odlind 2003; Truong et al. 2006; Francis and Jejeebhoy 2015	
Unplanned pregnancy	Spence, Elgen, and Harwell 2003; Curtis and Westoff et al. 1996; Campo et al. 2012	Borges et al. 2018
Partner support for FP	Ezeanolue et al. 2015; Agha 2010; Abraha, Belay, and Welay 2018; Triuneh et al. 2016; Onono et al. 2014; Yan et al. 2012; Hiltabiddle 1996; Akelo et al. 2013; Williams et al. 2008; Esber et al. 2014	
Married/partnered	Baumgarter et al. 2012; Newman et al. 2005; Akelo et al. 2013; Luo et al. 2018; Tiruneh et al. 2016; Legardy et al. 2005	Cavallaro et al. 2017 Essien et al. 2006
Type of relationship/sexual frequency	Irani, Speizer, and Fotso 2014; Luo et al. 2018; Spence, Elgen, and Harwell 2003; Campo et al. 2012; Francis and Jejeebhoy 2015	Yan et al. 2012
Parity	Adegbola and Okunowo 2009; Rink et al. 2012; Rai et al. 2017; Mogilevkina and Odlind 2003; Babalola et al. 2015	Picavet, Vlugt, and Wijsen 2014
Age	Adegbola and Okunowo 2009; Syum et al. 2019; Hamid, Stephenson, and Rubenson 2011; Nangia et al. 2003	Solanke, Oyinloye, and Asa 2018; Kuang, Ross, and Madsen2014; Spence, Elgen, and Harwell 2003
household decision making	Syum et al. 2019; Hamid, Stephenson, and Rubenson 2011; Rahman, Mostofa, and Hoque 2014; Mboane and Bhatta 2015	
Ideal family size	Kuang, Ross, and Madsen 2014; Tiruneh et al. 2016	
RuraL		Solanke, Oyinloye, and Asa 2018; Kuang, Ross, and Madsen 2014
Income	Kuang, Ross, and Madsen 2014; Tiruneh et al. 2016	
High‐risk pregnancy	French, Albanese, and Gossett 2016	
Late marriage	Tiruneh et al. 2016	
Breastfeeding		Udomboso et al. 2015; Fuell, Tossone, and Furman 2017
Violence	Murshid 2017	
Urban	Cavallaro et al. 2017	
Behavioral factors		
Attitude to contraception (willingness to use)	Wang et al. 2008; Raine et al. 2011; Hitabiddle et al. 1996; Lee, Carvallo, and Lee 2015; Syum et al. 2019; Picavet, Vlugt, and Wijsen 2014; Krenn et al. 2014; Mollen et al. 2008; Potard et al. 2012; Fuell, Tossone, and Furman 2017; Wang et al. 2004; Fazekas, Senn, and Ledgerwood 2001; O'Connor, Ferguson, and O'Connor 2005; Kang and Moneyham 2008; Lee et al. 2011; Gebremariam and Addissie 2014; Meskele et al. 2014; DeMaria et al. 2019	
Subjective norms (pressure from others) partner parents	Raine et al. 2011; Hitabiddle et al. 1996; Heeran et al. 2009; Khan et al. 2015; Lee, Carvallo, and Lee 2015; Rosengard et al. 2005; Akelo et al. 2013; Mollen et al. 2008; Williams et al. 2008; Gagnon and. Godin 2000; Esber et al. 2014; Abraha, Belay, and Welay 2018; Roderique‐Davies et al. 2016; Wang et al. 2004; Agha 2010; Babalola et al. 2015	
Knowledge of method	Shongwe, Ntuli, and Madiba 2019; Cavallaro et al. 2017; Tang et al. 2016; Khan et al. 2015; Syum et al. 2019; Abraha, Belay, and Welay 2018; Mesheriako et al. 2018; Moore et al. 2019; Larsson et al. 2004; Mogilevkina and Odlind 2003; Di Giacomo et al. 2013; Babalola et al. 2015; Gebremariam and Addissie 2014; Nguyen, Saucier, and Pica 1996; Khanal et al. 2011; Meskele et al. 2014; Costenbader et al. 2019	Newman et al. 2005; van der Westhuizen and Hanekom 2016
Previous experience	Joffe and Radius 1993; Hitabiddle et al. 1996; Huda et al. 2019; Picavet, Vlugt, and Wijsen 2014; Rosengard et al. 2005; Akelo et al. 2013; Esber et al. 2014; Luo et al. 2018; Adelman, Free, and Smith 2019; Ochejele et al. 2019; Kvalem et al. 2000; Campo et al. 2012; Wuni, Turpin, and Dassah 2017	
Perceived risk	Hitabiddle et al. 1996; Huda et al. 2019, Rosengard et al. 2005; Hoefnagels et al. 2006; Gagnon and Godin 2000; Elweshahi et al. 2018; Luo et al. 2018	
Perceived control	Mollen et al. 2008; Potard et al. 2012; Roderique‐Davies et al. 2016; DeMaria et al. 2019; Hanson et al. 2015	
Pregnancy intentions	Raine et al. 2011; Baumgarter et al. 2012; Rosengard et al. 2005; Luo et al. 2018; Spence, Elgen, and Harwell 2003; Gebremariam and Addissie 2014; Wuni, Turpin, and Dassah 2017	
Self‐efficacy	Shelus et al. 2018; Wang et al. 2008; Gagnon and Godin 2000; Wang et al. 2004; Cha, Kim, and Doswell 2007; Babalola et al. 2015	
Communication/discussion	Akelo et al. 2013; Shelus et al. 2018; Agha and Van Rossem 2002; Campo et al. 2012; Wuni, Turpin, and Dassah 2017; Agha 2001; Wang et al. 2004	
Attitude to pregnancy	Picavet, Vlugt, and Wijsen 2014; Akelo et al. 2013; Rai et al. 2017; Agha 2010.	
Injunctive norms/ social norms	Shongwe, Ntuli, and Madiba 2019; Costenbader et al. 2019	
What other people are doing	Mollen et al. 2008; Wang et al. 2004; Fazekas, Senn, and Ledgerwood 2001; Nguyen, Saucier, and Pica 1996	
Knowledge of use	Essien et al. 2006; Heeran et al. 2009	
Confidence/ease of use	Hitabiddle et al. 1996; Lee, Carvallo, and Lee 2015; Huda et al. 2019; Ochejele et al. 2019	
Anticipatory regret	Gagnon and. Godin 2000	
Perceived benefits	Roderique‐Davies et al. 2016	
Convenience	Hitabiddle et al. 1996; Heeran et al. 2009	
Personal norm	Williams et al. 2008	
Social identity	DeMaria et al. 2019	

A3. PAPERS USED IN THE REVIEW

Abraha, Teklehaymanot Huluf
, 
Hailay Siyum
Belay
, and 
Getachew Mebrahtu
Welay
. 2018. “Intentions on Contraception Use and Its Associated Factors among Postpartum Women in Aksum Town, Tigray Region, Northern Ethiopia: A Community‐Based Cross‐sectional Study 11 Medical and HEALTH sciences 1117 Public Health and Health Services.” Reproductive Health
15(1): 188. 10.1186/s12978-018-0632-2.30413214PMC6234798

Adegbola, Omololu
, and 
Adeyemi
Okunowo
. 2009. “Intended Postpartum Contraceptive Use among Pregnant and Puerperal Women at a University Teaching Hospital.” Archives of Gynecology and Obstetrics
280(6): 987–992. 10.1007/s00404-009-1056-6.19322573

Adelman, Sara
, 
Caroline
Free
, and 
Chris
Smith
. 2019. “Predictors of Postabortion Contraception Use in Cambodia.” Contraception
99(3): 155–159. 10.1016/j.contraception.2018.11.010.30471264

Agha, S.

2001. “Intention to Use the Female Condom Following a Mass‐Marketing Campaign in Lusaka, Zambia.” American Journal of Public Health
91(2): 307–310. 10.2105/AJPH.91.2.307.11211646PMC1446544

Agha, Sohail
. 2010. “Intentions to Use Contraceptives in Pakistan: Implications for Behavior Change Campaigns.” BMC Public Health
10: 450. 10.1186/1471-2458-10-450.20673374PMC2920282

Agha, Sohail
, and 
Ronan
Van Rossem
. 2002. “Impact of Mass Media Campaigns on Intentions to Use the female condom in Tanzania.” International Family Planning Perspectives
28(3): 151–158. 10.2307/3088258.

Akelo, Victor
, 
Sonali
Girde
, 
Craig B.
Borkowf
, 
Frank
Angira
, 
Kevin
Achola
, 
Richard
Lando
, 
Lisa A.
Mills
, 
Timothy K.
Thomas
, and 
Shirley Lee
Lecher
. 2013. “Attitudes toward Family Planning among HIV‐Positive Pregnant Women Enrolled in a Prevention of Mother‐to‐Child Transmission Study in Kisumu, Kenya.” PLoS ONE
8(8): e66593. 10.1371/journal.pone.0066593.23990868PMC3753279

Amin, Ruhul
, and 
Takanori
Sato
. 2004. “Impact of a School‐Based Comprehensive Program for Pregnant Teens on their Contraceptive Use, Future Contraceptive Intention, and Desire for More Children.” Journal of Community Health Nursing
21(1): 39–47. 10.1207/s15327655jchn2101_4.14979845

Atiglo, D. Yaw
, and 
Samuel N.A.
Codjoe
. 2019. “Meeting Women's Demand for Contraceptives in Ghana: Does Autonomy Matter?” Women and Health
59(4): 347–363. 10.1080/03630242.2018.1500413.30040604

Babalola, Stella
, 
Neetu
John
, 
Bolanle
Ajao
, and 
Ilene S.
Speizer
. 2015. “Ideation and Intention to Use Contraceptives in Kenya and Nigeria.” Demographic Research
33(1): 211–238. 10.4054/DemRes.2015.33.8.31303859PMC6625811

Barden‐O'Fallon, Janine
, 
Jennifer
Mason
, 
Emmanuel
Tluway
, 
Gideon
Kwesigabo
, and 
Egidius
Kamanyi
. 2020. “Counseling on Injectable Contraception and HIV Risk: Evaluation of a Pilot Intervention in Tanzania.” PLoS One
15(4): e0231070. 10.1371/journal.pone.0231070.32243478PMC7122807

Baumgartner, Joy Noel
, 
Rose
Otieno‐Masaba
, 
Mark A.
Weaver
, 
Thomas W.
Grey
, and 
Heidi W.
Reynolds
. 2012. “Service Delivery Characteristics Associated with Contraceptive Use among Youth Clients in Integrated Voluntary Counseling and HIV Testing Clinics in Kenya.” AIDS Care ‐ Psychological and Socio‐Medical Aspects of AIDS/HIV
24(10): 1290–1301. 10.1080/09540121.2012.658753.22435668

Borges, Ana Luiza


Vilela, Osmara


Alves dos
Santos
, and 
Elizabeth
Fujimori
. 2018. “Concordance between Intention to Use and Current Use of Contraceptives among Six‐Month Postpartum Women in Brazil: The Role of Unplanned Pregnancy.” Midwifery
56: 94–101. 10.1016/j.midw.2017.10.015.29096285

Brown, Katherine E.
, 
Keith M.
Hurst
, and 
Madelynne A.
Arden
. 2011. “Improving Adolescent Contraceptive Use: Evaluation of a Theory‐Driven Classroom‐Based Intervention.” Psychology, Health and Medicine
16(2): 141–155. 10.1080/13548506.2010.525791.21328143

Bryant, Amy G
, 
Ilene S
Speizer
, 
Jennifer C
Hodgkinson
, 
Alison
Swiatlo
, 
Siân L
Curtis
, and 
Krista
Perreira
. 2018. “Contraceptive Practices, Preferences, and Barriers among Abortion Clients in North Carolina.” Southern Medical Journal
111(6): 317–323. 10.14423/SMJ.0000000000000820.29863217PMC5989576

Callahan, Rebecca L.
, and 
Stan
Becker
. 2014. “Unmet Need, Intention to Use Contraceptives and Unwanted Pregnancy in Rural Bangladesh.” International Perspectives on Sexual and Reproductive Health
40(1): 4–10. 10.1363/4000414.24733056

Campo, Shelly
, 
Natoshia M.
Askelson
, 
Erica L.
Spies
, and 
Mary
Losch
. 2012. “Ambivalence, Communication and Past Use: Understanding What Influences Women's Intentions to Use Contraceptives.” Psychology, Health and Medicine
17(3): 356–365. 10.1080/13548506.2011.608432.21895569

Cavallaro, Francesca L
, 
Lenka
Benova
, 
David
Macleod
, 
Adama
Faye
, and 
Caroline A
Lynch
. 2017. “Examining Trends in Family Planning among Harder‐to‐Reach Women in Senegal 1992–2014.” Scientific Reports
7: 41006. 10.1038/srep41006.28106100PMC5247687

Cha, Eun Seok
, 
Kevin H.
Kim
, and 
Willa M.
Doswell
. 2007. “Influence of the Parent‐Adolescent Relationship on Condom Use among South Korean Male College Students.” Nursing and Health Sciences
9(4): 277–283. 10.1111/j.1442-2018.2007.00326.x.17958677

Costenbader
E
, 
Zissette
S
, 
Martinez
A
, 
LeMasters
K
, 
Dagadu
NA
, 
Deepan
P
, et al. 2019. “Getting to Intent: Are Social Norms Influencing Intentions to Use Modern Contraception in the DRC?”
PLoS One
14(7): e0219617. 10.1371/journal.pone.0219617
31310641PMC6634398

Curtis, S L
, and 
C F
Westoff
. 1996. “Intention to Use Contraceptives and Subsequent Contraceptive Behavior in Morocco. Studies in Family Planning
27(5): 239–50.8923652

Di Giacomo, Patrizia
, 
Alessia
Sbarlati
, 
Annamaria
Bagnasco
, and 
Loredana
Sasso
. 2013. “Woman's Contraceptive Needs and Preferences in the Postpartum Period: An Italian Study.” Journal of Clinical Nursing
22(23–24): 3406–3417. 10.1111/jocn.12432.24580788

DeMaria, A. L.
, 
Sundstrom, B.
, 
Faria, A. A.
, 
Moxley Saxon, G.
, and 
Ramos‐Ortiz, J.

2019. “Using the Theory of Planned Behavior and Self‐Identity to Explore Women's Decision‐Making and Intention to Switch from Combined Oral Contraceptive Pill (COC) to Long‐Acting Reversible Contraceptive (LARC). BMC Women's Health
19(1): 82. 10.1186/s12905-019-0772-8
31221144PMC6585137

Eliason, Sebastian
, 
Frank
Baiden
, 
Derek Anamaale
Tuoyire
, and 
Kofi
Awusabo‐Asare
. 2018. “Sex Composition of Living Children in a Matrilineal Inheritance System and Its Association with Pregnancy Intendedness and Postpartum Family Planning Intentions in Rural Ghana.” Reproductive Health
15(1): 187. 10.1186/s12978-018-0616-2.30413219PMC6234793

Elweshahi, Heba Mahmoud Taha
, 
Gihan Ismail
Gewaifel
, 
Sameh Saad
EL‐Din Sadek
, and 
Omnia Galal
El‐Sharkawy
. 2018. “Unmet Need for Postpartum Family Planning in Alexandria, Egypt.” Alexandria Journal of Medicine
54(2): 143–147. 10.1016/j.ajme.2017.03.003.

Esber, Allahna
, 
Randi E.
Foraker
, 
Maryam
Hemed
, and 
Alison
Norris
. 2014. “Partner Approval and Intention to Use Contraception among Zanzibari Women Presenting for Post‐Abortion Care.” Contraception
90(1): 23–28. 10.1016/j.contraception.2014.03.006.24809805

Essien, Ekere James
, 
Gbadebo O
Ogungbade
, 
Harrison N
Kamiru
, 
Ernest
Ekong
, 
Doriel
Ward
, and 
Laurens
Holmes
. 2006. “Emerging Sociodemographic and Lifestyle Predictors of Intention to Use Condom in Human Immunodeficiency Virus Intervention among Uniformed Services Personnel.” Military Medicine
171(10): 1027–34. 10.7205/milmed.171.10.1027.17076460PMC1643840

Ezeanolue, Echezona E.
, 
Juliet
Iwelunmor
, 
Ibitola
Asaolu
, 
Michael C.
Obiefune
, 
Chinenye O.
Ezeanolue
, 
Alice
Osuji
, 
Amaka G.
Ogidi
, et al. 2015. “Impact of Male Partner's Awareness and Support for Contraceptives on Female Intent to Use Contraceptives in Southeast Nigeria Health Behavior, Health Promotion and Society.” BMC Public Health
15: 879. 10.1186/s12889-015-2216-1.26358642PMC4566290

Fazekas, Anna
, 
Charlene Y.
Senn
, and 
David M.
Ledgerwood
. 2001. “Predictors of Intention to Use Condoms among University Women: An Application and Extension of the Theory of Planned Behaviour.” Canadian Journal of Behavioural Science
33(2): 103–117. 10.1037/h0087133.

French, Maureen
, 
Alexandra
Albanese
, and 
Dana R.
Gossett
. 2016. “Postpartum Contraceptive Choice after High‐Risk Pregnancy: A Retrospective Cohort Analysis.” Contraception
94(2): 173–180. 10.1016/j.contraception.2016.04.004.27091723

Fuell Wysong, Elena
, 
Krystel
Tossone
, and 
Lydia
Furman
. 2017. “Expectant Inner‐City Women: Attitudes about Contraception Given Infant Feeding Choice.” European Journal of Contraception and Reproductive Health Care
22(5): 369–374. 10.1080/13625187.2017.1397110.29131703

Gagnon, Marie Pierre
, and 
Gaston
Godin
. 2000. “The Impact of New Antiretroviral Treatments on College Students’ Intention to Use a Condom with a New Sexual Partner.” AIDS Education and Prevention
12(3): 239–251.10926127

Gebremariam, Alem
, and 
Adamu
Addissie
. 2014. “Intention to Use Long Acting and Permanent Contraceptive Methods and Factors Affecting It among Married Women in Adigrat Town, Tigray, Northern Ethiopia.” Reproductive Health
11: 24. 10.1186/1742-4755-11-24.24628764PMC4007570

Graham, Anna
, 
Laurence
Moore
, 
Deborah
Sharp
, and 
Ian
Diamond
. 2002. “Improving Teenagers’ Knowledge of Emergency Contraception: Cluster Randomised Controlled Trial of a Teacher Led Intervention.” British Medical Journal
324(7347): 1179–1183. 10.1136/bmj.324.7347.1179.12016180PMC111106

Grimley, DM
, and 
PA
Lee
. 1997. “Condom and Other Contraceptive Use among a Random Sample of Female Adolescents: A Snapshot in Time.” Adolescence
32(128): 771–9.9426803

Gupta, Neeru
, 
Charles
Katende
, and 
Ruth E.
Bessinger
. 2003. “Associations of Mass Media Exposure with Family Planning Attitudes and Practices in Uganda.” Studies in Family Planning
34(1): 19–31. 10.1111/j.1728-4465.2003.00019.x.12772443

Haile, Kebede
, 
Meresa
Gebremedhin
, 
Haileselasie
Berhane
, 
Tirhas
Gebremedhin
, 
Alem
Abraha
, 
Negassie
Berhe
, 
Tewodros
Haile
, 
Goitom
Gigar
, and 
Yonas
Girma
. 2016. “Desire for Birth Spacing or Limiting and Non‐use of Long Acting and Permanent Contraceptive Methods among Married Women of Reproductive Age in Aksum Town, North Ethiopia.” Contraception and Reproductive Medicine
1: 22. 10.1186/s40834-016-0033-2.29201411PMC5693538

Hamid, Saima
, 
Rob
Stephenson
, and 
Birgitta
Rubenson
. 2011. “Marriage Decision Making, Spousal Communication, and Reproductive Health among Married Youth in Pakistan.” Global Health Action
4: 5079. 10.3402/gha.v4i0.5079.21253456PMC3023879

Hanson, JD
, 
Nothwehr, F
, 
Yang, JG
, and 
Romitti, P.

2015. “Indirect and Direct Perceived Behavioral Control and the Role of Intention in the Context of Birth Control Behavior.” Materna; and Child Health Journal
19(7): 1535–1542. 10.1007/s10995-014-1658-x.PMC589632525421330

Heeren, G Anita
, 
John B
Jemmott
, 
Andrew
Mandeya
, and 
Joanne C
Tyler
. 2009. “Sub‐Saharan African University Students’ Beliefs about Condoms, Condom‐Use Intention, and Subsequent Condom Use: A Prospective Study.” AIDS and Behavior
13(2): 268–276. 10.1007/s10461-008-9415-z.18600442PMC4349680

Hiltabiddle, S. J.

1996. “Adolescent Condom Use, the Health Belief Model, and the Prevention of Sexually Transmitted Disease.” Journal of Obstetric, Gynecologic, and Neonatal Nursing
25(1): 61–66. 10.1111/j.1552-6909.1996.tb02514.x.8627404

Hoefnagels, Cees
, 
Harm J.
Hospers
, 
Clemens
Hosman
, 
Leo
Schouten
, and 
Herman
Schaalma
. 2006.“ One Measure, Two Motives. Prediction of Condom Use and Interaction between Two Prevention Goals among Heterosexual Young Adults: Preventing Pregnancy and/or Sexually Transmitted Diseases.” Prevention Science
7(4): 369–376. 10.1007/s11121-006-0048-z.16823634

Huda, Fauzia Akhter
, 
John B.
Casterline
, 
Faisal
Ahmmed
, 
Kazuyo
Machiyama
, 
Hassan
Rushekh Mahmood
, 
Anisuddin
Ahmed
, and 
John
Cleland
. 2019. “Contraceptive Method Attributes and Married Women's Intention to Use the Pill or the Injectable in Rural Bangladesh.” International Perspectives on Sexual and Reproductive Health
44(4): 157–165. 10.1363/44e7118.31381499

Irani, Laili
, 
Ilene S
Speizer
, and 
Jean‐Christophe
Fotso
. 2014. “Relationship Characteristics and Contraceptive Use among Couples in Urban Kenya.” International Perspectives on Sexual and Reproductive Health
40(1): 11–20. 10.1363/4001114.24733057PMC4317354

Jawad, Aalaa
, 
Issrah
Jawad
, and 
Nisreen A.
Alwan
. 2019. “Interventions Using Social Networking Sites to Promote Contraception in Women of Reproductive Age.” Cochrane Database of Systematic Reviews (3). Art. No.: CD01252. John Wiley and Sons. 10.1002/14651858.CD012521.pub2.PMC639522530818414

Joffe, A
, and 
S M
Radius
. 1993. “Self‐Efficacy and Intent to Use Condoms among Entering College Freshmen.” The Journal of Adolescent Health
14(4): 262–268. 10.1016/1054-139x(93)90172-l.8347636

Johnson, Natasha A
, 
Elena Fuell
Wysong
, 
Krystel
Tossone
, and 
Lydia
Furman
. 2019. “Associations between Prenatal Intention and Postpartum Choice: Infant Feeding and Contraception Decisions among Inner‐City Women.” Breastfeeding Medicine
14(7): 456–464. 10.1089/bfm.2018.0248.31166698

Kang, Hee Sun
, and 
Linda
Moneyham
. 2008. “Use of Emergency Contraceptive Pills and Condoms by College Students: A Survey.” International Journal of Nursing Studies
45(5): 775–783. 10.1016/j.ijnurstu.2007.01.008.17349645

Kgosiemang, Bobby
, and 
Julia
Blitz
. 2018. “Emergency Contraceptive Knowledge, Attitudes and Practices among Female Students at the University of Botswana: A Descriptive Survey.” African Journal of Primary Health Care & Family Medicine
10(1): e1–e6. 10.4102/phcfm.v10i1.1674.PMC613169530198288

Khan, Mishal S.
, 
Farah Naz
Hashmani
, 
Owais
Ahmed
, 
Minaal
Khan
, 
Sajjad
Ahmed
, 
Shershah
Syed
, and 
Fahad
Qazi
. 2015. “Quantitatively Evaluating the Effect of Social Barriers: A Case‐Control Study of Family Members’ Opposition and Women's Intention to Use Contraception in Pakistan.” Emerging Themes in Epidemiology
12: 2. 10.1186/s12982-015-0023-x.25642278PMC4312593

Khan, Sadaf
, 
Breanne
Grady
, and 
Sara
Tifft
. 2015. “Estimating Demand for a New Contraceptive Method: Projections for the Introduction of Sayana Press.” International Journal of Gynecology and Obstetrics
130: E21–E24. 10.1016/j.ijgo.2015.03.020.26092777

Khanal, V.
, 
C.
Joshi
, 
D.
Neupane
, and 
R.
Karkee
. 2011. “Practices and Perceptions on Contraception Acceptance among Clients Availing Safe Abortion Services in Nepal.” Kathmandu University Medical Journal
9(35): 179–184. 10.3126/kumj.v9i3.6301.22609503

Kim, Hae Won
. 2015. “Sex Differences in the Awareness of Emergency Contraceptive Pills Associated with Unmarried Korean University Students’ Intention to Use Contraceptive Methods: An Online Survey.” Reproductive Health
12(1): 91. 10.1186/s12978-015-0076-x.26395172PMC4580124

Klufio, CA
, 
AB
Amoa
, and 
G
Kariwiga
. “A Survey of Papua New Guinean Parturients at the Port Moresby General Hospital: Family Planning.” Journal of Biosocial Science
27(1), (January 1995): 11–8. 10.1017/s0021932000006969.7876291

Krenn, Susan
, 
Lisa
Cobb
, 
Stella
Babalola
, 
Mojisola
Odeku
, and 
Bola
Kusemiju
. 2014. “Using Behavior Change Communication to Lead a Comprehensive Family Planning Program: The Nigerian Urban Reproductive Health Initiative’ Global Health,” Science and Practice
2(4): 427–443. 10.9745/GHSP-D-14-00009.PMC430785925611477

Kuang, Bernice
, 
John
Ross
, and 
Elizabeth Leahy
Madsen
. 2014. “Defining Motivational Intensity of Need for Family Planning in Africa.” African Journal of Reproductive Health
18(3): 57–66.25438510

Kvalem, Ingela Lundin
, and 
Bente
Træen
. 2000. “Self‐Efficacy, Scripts of Love and Intention to Use Condoms among Norwegian Adolescents.” Journal of Youth and Adolescence
29(3): 337–353. 10.1023/A:1005199725666.

Larsson, Margareta
, 
Karin
Eurenius
, 
Ragnar
Westerling
, and 
Tanja
Tydén
. 2004. “Emergency Contraceptive Pills in Sweden: Evaluation of an Information Campaign.” BJOG: An International Journal of Obstetrics and Gynaecology
111(8): 820–827. 10.1111/j.1471-0528.2004.00206.x.15270930

Lee, Jongwon
, 
Mauricio
Carvallo
, and 
Taehun
Lee
. 2015. “Psychometric Properties of a Measure Assessing Attitudes and Norms as Determinants of Intention to Use Oral Contraceptives.” Asian Nursing Research
9(2): 138–145. 10.1016/j.anr.2015.04.003.26160243

Lee, Jongwon
, 
Mary Ann
Jezewski
, 
Yow Wu Bill
Wu
, and 
Mauricio
Carvallo
. 2011. “The Relationship between Acculturation and Oral Contraceptive Use among Korean Immigrant Women.” Research in Nursing and Health
34(2): 91–102. 10.1002/nur.20417.21381043

Legardy, Jennifer K.
, 
Maurizio
Macaluso
, 
Lynn
Artz
, and 
Ilene
Brill
. “Do Participant Characteristics Influence the Effectiveness of Behavioral Interventions? Promoting Condom Use to Women.” Sexually Transmitted Diseases
32(11) (2005): 665–671. 10.1097/01.olq.0000175392.84989.ec.16254540

Lori, Jody R.
, 
Meagan
Chuey
, 
Michelle L.
Munro‐Kramer
, 
Henrietta
Ofosu‐Darkwah
, and 
Richard M.K.
Adanu
. 2018. “Increasing Postpartum Family Planning Uptake through Group Antenatal Care: A Longitudinal Prospective Cohort Design.” Reproductive Health
15: 208. 10.1186/s12978-018-0644-y.30558677PMC6296041

Luo, Zhongchen
, 
Lingling
Gao
, 
Ronald
Anguzu
, and 
Juanjuan
Zhao
. 2018. “Long‐Acting Reversible Contraceptive Use in the Post‐Abortion Period among Women Seeking Abortion in Mainland China: Intentions and Barriers.” Reproductive Health
15: 85. 10.1186/s12978-018-0543-2.29793501PMC5968602

Lutalo, Tom
, 
Ron
Gray
, 
John
Santelli
, 
David
Guwatudde
, 
Heena
Brahmbhatt
, 
Sanyukta
Mathur
, 
David
Serwadda
, 
Fred
Nalugoda
, and 
Fredrick
Makumbi
. 2018. “Unfulfilled Need for Contraception among Women with Unmet Need but with the Intention to Use Contraception in Rakai, Uganda: A Longitudinal Study.” BMC Women's Health
18: 60. 10.1186/s12905-018-0551-y.29699548PMC5921782

Mboane, Ramos
, and 
Madhav P.
Bhatta
. 2015. “Influence of a Husband's Healthcare Decision Making Role on a Woman's Intention to Use Contraceptives among Mozambican Women.” Reproductive Health
12: 36. 10.1186/s12978-015-0010-2.25902830PMC4409755

McCarthy, Ona L.
, 
Hanadi
Zghayyer
, 
Amina
Stavridis
, 
Samia
Adada
, 
Irrfan
Ahamed
, 
Baptiste
Leurent
, 
Phil
Edwards
, 
Melissa
Palmer
, and 
Caroline
Free
. 2019. “A Randomized Controlled Trial of an Intervention Delivered by Mobile Phone Text Message to Increase the Acceptability of Effective Contraception among Young Women in Palestine.” Trials
20: 228. 10.1186/s13063-019-3297-4.31014358PMC6477750

Mekonnen, Getachew
, 
Fikre
Enquselassie
, 
Gezahegn
Tesfaye
, and 
Agumasie
Semahegn
. 2014. “Prevalence and Factors Affecting Use of Long Acting and Permanent Contraceptive Methods in Jinka Town, Southern Ethiopia: A Cross sectional Study.” Pan African Medical Journal
18: 98. 10.11604/pamj.2014.18.98.3421.25404960PMC4232023

Mesfin, Yonatan Moges
, and 
Kelemu
Tilahun
. 2016. “Practice and Intention to Use Long Acting and Permanent Contraceptive Methods among Married Women in Ethiopia: Systematic Meta‐Analysis.” Reproductive Health
13: 78. 10.1186/s12978-016-0194-0.27329147PMC4915059

Mogilevkina, I.
, and 
V.
Odlind
. 2003. “Contraceptive Practices and Intentions of Ukrainian Women.” The European Journal of Contraception & Reproductive Health Care
8(4): 185–196. 10.1080/713604469.15006265

Mollen, Cynthia J
, 
Frances K.
Barg
, 
Katie L.
Hayes
, 
Marah
Gotcsik
, 
Nakeisha M
Blades
, and 
Donald F.
Schwarz
. 2008. “Assessing Attitudes about Emergency Contraception among Urban, Minority Adolescent Girls: An In‐Depth Interview Study.” Pediatrics
122(2): e395–401. 10.1542/peds.2008-0009.18676526

Moore, Crystal L.
, 
Alison H.
Edie
, 
Jennifer L.
Johnson
, and 
Eleanor L.
Stevenson
. 2019. “Long‐Acting Reversible Contraception: Assessment of Knowledge and Interest among College Females.” Journal of American College Health
67(7): 615–619. 10.1080/07448481.2018.1500473.30239327

Murshid, Nadine Shaanta
. 2017. “Intimate Partner Violence and Contraception in Pakistan: Results from Pakistan Demographic and Health Survey 2012–13.” Women's Studies International Forum
64: 10–16. 10.1016/j.wsif.2017.08.003.

Newmann, Sara J.
, 
Daniel
Grossman
, 
Cinthia
Blat
, 
Maricianah
Onono
, 
Rachel
Steinfeld
, 
Elizabeth
A. Bukusi
, 
Starley
Shade
, and 
Craig
R. Cohen
. “Does Integrating Family Planning into HIV Care and Treatment Impact Intention to Use Contraception? Patient Perspectives from HIV‐Infected Individuals in Nyanza Province, Kenya.” International Journal of Gynecology and Obstetrics
123(suppl 1), (November 2013). 10.1016/j.ijgo.2013.08.001.PMC384236524008310

Nguyen, Minh Nguyet
, 
Jean François
Saucier
, and 
Lucille A.
Pica
. 1996. “Factors Influencing the Intention to Use Condoms in Quebec Sexually‐Inactive Male Adolescents.” Journal of Adolescent Health
18(1): 48–53. 10.1016/1054-139X(95)00045-T.8750428

Ochejele, Silas
, 
Chris
Ega
, 
Muhammad Shakir
Balogun
, 
Patrick
Nguku
, 
Tunde
Adedokun
, and 
Hadiza
Usman
. 2019. “Contraceptive Intentions of Survivors of Severe Acute Maternal Morbidity in Kaduna State, Nigeria.” European Journal of Contraception and Reproductive Health Care
24(2): 161–165. 10.1080/13625187.2019.1569222.30920324

O'Connor, Daryl B.
, 
Eamonn
Ferguson
, and 
Rory C.
O'Connor
. 2005. “Intentions to Use Hormonal Male Contraception: The Role of Message Framing, Attitudes and Stress Appraisals.” British Journal of Psychology
96(3): 351–369. 10.1348/00071260549114.16131412

Onono, Maricianah
, 
Cinthia
Blat
, 
Sondra
Miles
, 
Rachel
Steinfeld
, 
Pauline
Wekesa
, 
Elizabeth A.
Bukusi
, 
Kevin
Owuor
, 
Daniel
Grossman
, 
Craig R.
Cohen
, and 
Sara J.
Newmann
. 2014. “Impact of Family Planning Health Talks by lay Health Workers on Contraceptive Knowledge and Attitudes among HIV‐Infected Patients in Rural Kenya.” Patient Education and Counseling
94(3): 438–441. 10.1016/j.pec.2013.11.008.24316053PMC4530318

Peterson, Sara Ann
. 1999. “Marriage Structure and Contraception in Niger.” Journal of Biosocial Science
31(1): 93–104. 10.1017/S0021932099000930.10081240

Picavet, Charles
, 
Ineke
van der
Vlugt
, and 
Ciel
Wijsen
. 2014. “Intention to Use Emergency Contraceptive Pills and the Role of Knowledge in a Dutch National Sample.” The European Journal of Contraception & Reproductive Health Care
19(4): 250–258. 10.3109/13625187.2014.910595.24856152

Potard, Catherine
, 
Robert
Courtois
, 
Mathieu Le
Samedy
, 
B
Mestre
, 
M J
Barakat
, and 
Christian
Réveillère
. 2012. “Determinants of the Intention to Use Condoms in a Sample of French Adolescents.” The European Journal of Contraception & Reproductive Health Care
17(1): 55–64. 10.3109/13625187.2011.634455.22149900

Rahman, Md Mosfequr
, 
Md Golam
Mostofa
, and 
Md Aminul
Hoque
. 2014. “Women's Household Decision‐Making Autonomy and Contraceptive Behavior among Bangladeshi Women.” Sexual and Reproductive Healthcare
5(1): 9–15. 10.1016/j.srhc.2013.12.003.24472384

Rai, Rajesh Kumar
. 2017. “Do the Sex Composition of living children and the desire for additional children affect future intention to use contraception in Ethiopia?” Journal of Biosocial Science
49(6): 757–772. 10.1017/S0021932016000729.28069079

Rai, Rajesh Kumar
, and Sayeed Unisa. “Dynamics of Contraceptive Use in India: Apprehension versus Future Intention among Non‐users and Traditional Method Users.” Sexual and Reproductive Healthcare
4(2) (2013): 65–72. 10.1016/j.srhc.2013.03.001.23663924

Raine, Tina R
, 
Anne
Foster‐Rosales
, 
Ushma D
Upadhyay
, 
Cherrie B
Boyer
, 
Beth A
Brown
, 
Abby
Sokoloff
, and 
Cynthia C
Harper
. 2011. “One‐Year Contraceptive Continuation and Pregnancy in Adolescent Girls and Women Initiating Hormonal Contraceptives.” Obstetrics and Gynecology
117(2, Pt 1): 363–371. 10.1097/AOG.0b013e31820563d3.21252751PMC3154007

Raine‐Bennett, Tina R.
, and 
Corinne H.
Rocca
. 2015. “Development of a Brief Questionnaire to Assess Contraceptive Intent.” Patient Education and Counseling
98(11): 1425–1430. 10.1016/j.pec.2015.05.016.26104994PMC4609226

Reeves, Matthew F.
, 
Qiuhong
Zhao
, 
Gina M.
Secura
, and 
Jeffrey F.
Peipert
. 2016. “Risk of Unintended Pregnancy Based on Intended Compared to Actual Contraceptive Use.” American Journal of Obstetrics and Gynecology
215(1): 71.e1–71.e6. 10.1016/j.ajog.2016.01.162.26805610

Rink, Elizabeth
, 
Kris
FourStar
, 
Jarrett Medicine
Elk
, 
Rebecca
Dick
, 
Lacey
Jewett
, and 
Dionne
Gesink
. 2012. “Pregnancy Prevention among American Indian Men Ages 18 to 24: The Role of Mental Health and Intention to Use Birth Control.” American Indian and Alaska Native Mental Health Research
19(1): 57–75. 10.5820/aian.1901.2012.57.22569725

Rink, Elizabeth
, 
Kris
FourStar
, 
Jarrett Medicine
Elk
, 
Rebecca
Dick
, 
Lacey
Jewett
, and 
Dionne
Gesink
. 2012. “Young Native American Men and Their Intention to Use Family Planning Services.” American Journal of Men's Health
6(4): 324–330. 10.1177/1557988312439226.22433656

Roderique‐Davies, Gareth
, 
Christine
McKnight
, 
Bev
John
, 
Susan
Faulkner
, and 
Deborah
Lancastle
. 2016. “Models of Health Behaviour Predict Intention to Use Long‐Acting Reversible Contraception.” Women's Health
12(6): 507–512. 10.1177/1745505716678231.PMC537325927864572

Rosengard, Cynthia
, 
Jennifer G
Clarke
, 
Kristen
DaSilva
, 
Megan
Hebert
, 
Jennifer
Rose
, and 
Michael D
Stein
. 2005. “Correlates of Partner‐Specific Condom Use Intentions among Incarcerated Women in Rhode Island.” Perspectives on Sexual and Reproductive Health
37(1): 32–38. 10.1363/psrh.37.32.05.15888401PMC1351210

Ross, John A.
, and 
William L.
Winfrey
. 2001. “Contraceptive Use, Intention to Use and Unmet Need during the Extended Postpartum Period.” International Family Planning Perspectives
27(1): 20–27. 10.2307/2673801.

Roy, T. K.
, 
F.
Ram
, 
Parveen
Nangia
, 
Uma
Saha
, and 
Nizamuddin
Khan
. 2003. “Can Women's Childbearing and Contraceptive Intentions Predict Contraceptive Demand? Findings from a Longitudinal Study in Central India.” International Family Planning Perspectives
29(1): 25–31. 10.2307/3180998.12709309

Sarnak, Dana Amy Tsui
, 
Fredrick
Makumbi
, 
Simon P. S
Kibira
, and 
Saifuddin
Ahmed
. 2020. “The Predictive Utility of Unmet Need on Time to Contraceptive Adoption: A Panel Study of Non‐Contracepting Ugandan Women,” Contraception: X
2: 100022. 10.1016/j.conx.2020.100022.32550537PMC7286181

Samanta, Sumitra Dhal
, 
Gulnoza
Usmanova
, 
Anjum
Shaheen
, 
Murari
Chandra
, and 
Sunil
Mehra
. 2018. “Family‐Centric Safe Motherhood Approach for Marginalized Young Married Couples in Rural India.” Journal of Family Medicine and Primary Care
7(5): 852–858. 10.4103/jfmpc.jfmpc_351_17.PMC625954430598923

Shelus, Victoria
, 
Lauren
VanEnk
, 
Monica
Giuffrida
, 
Stefan
Jansen
, 
Scott
Connolly
, 
Marie
Mukabatsinda
, 
Fatou
Jah
, 
Vedasta
Ndahindwa
, and 
Dominick
Shattuck
. 2018. “Understanding Your Body Matters: Effects of an Entertainment‐Education Serial Radio Drama on Fertility Awareness in Rwanda.” Journal of Health Communication
23(8): 761–772. 10.1080/10810730.2018.1527873.30289356

Shongwe, Philile
, 
Busisiwe
Ntuli
, and 
Sphiwe
Madiba
. 2019. “Assessing the Acceptability of Vasectomy as a Family Planning Option: A Qualitative Study with Men in the Kingdom of Eswatini.” International Journal of Environmental Research and Public Health
16(24). 10.3390/ijerph16245158.PMC695013231861151

Solanke, Bola Lukman
, 
Olufunmilola
Olufunmilayo Banjo
, 
Bosede Odunola
Oyinloye
, and 
Soladoye Sunday
Asa
. 2018. “Maternal Grand Multiparity and Intention to Use Modern Contraceptives in Nigeria.” BMC Public Health
18: 1207. 10.1186/s12889-018-6130-1.30373559PMC6206733

Spence, Michael R.
, 
Kindra K.
Elgen
, and 
Todd S.
Harwell
. 2003. “Awareness, Prior Use, and Intent to Use Emergency Contraception among Montana Women at the Time of Pregnancy Testing.” Maternal and Child Health Journal
7(3): 197–203. 10.1023/A:1025140522138.14509415

Syum, Hailay
, 
Gizienesh
Kahsay
, 
Teklehaymanot
Huluf
, 
Berhe
Beyene
, 
Hadgu
Gerensea
, 
Gebreamlak
Gidey
, 
Haftom
Desta
, 
Mebrahtu
Abay
, 
Haben
Nuguse
, and 
Kebede
Haile
. 2019. “Intention to Use Long‐Acting and Permanent Contraceptive Methods and Associated Factors in Health Institutions of Aksum Town, North Ethiopia.” BMC Research Notes
12: 739. 10.1186/s13104-019-4769-z.31706362PMC6842538

Tang, Jennifer H
, 
Dawn M
Kopp
, 
Gretchen S
Stuart
, 
Michele
O'Shea
, 
Christopher C
Stanley
, 
Mina C
Hosseinipour
, 
William C
Miller
, et al. 2016. “Association between Contraceptive Implant Knowledge and Intent with Implant Uptake among Postpartum Malawian Women: A Prospective Cohort Study.” Contraception and Reproductive Medicine
1(1): 13. 10.1186/s40834-016-0026-1.29201402PMC5693581

Tiruneh, Fentanesh Nibret
, 
Kun Yang
Chuang
, 
Peter A.M.
Ntenda
, and 
Ying Chih
Chuang
. 2016. “Factors Associated with Contraceptive Use and Intention to Use Contraceptives among Married Women in Ethiopia.” Women and Health
56(1): 1–22. 10.1080/03630242.2015.1074640.26212154

Truong, Hong


Ha
M.
, 
Timothy
Kellogg
, 
Willi
McFarland
, 
Mi Suk
Kang
, 
Philip
Darney
, and 
Eleanor A.
Drey
. 2006. “Contraceptive Intentions among Adolescents after Abortion.” Journal of Adolescent Health
39(2): 283–286. 10.1016/j.jadohealth.2005.11.025.16857542

Udombosoa, Christopher


Godwin, A. Y. Amoatenga
, and 
P. T.
Doegah
. 2015. Biosocial correlates of intention to use or not to use contraception: The case of Ghana and Nigeria. African Population Studies
29(2): 2081–2100.

Unger, Jennifer B.
, and 
Gregory
B. Molina
. “Contraceptive Use among Latina Women: Social, Cultural, and Demographic Correlates.” Women's Health Issues
8(6) (November 1998): 359–369. 10.1016/S1049-3867(98)00030-9.9846120

Valencia, Claudia P
, and 
Gladys E
Canaval
. 2012. “Factores que predisponen, facilitan y refuerzan el uso del preservativo en jóvenes universitarios de Cali, Colombia” [Factors predisposing, facilitating and strengthening condom use amongst university students in Cali, Colombia]. Revista de Salud Publica (Bogota, Colombia)
14(5): 810–821.24652360

Vroome, de EM
, 
ME
Paalman
, 
TG
Sandfort
, 
M
Sleutjes
, 
KJ
de Vries
, and 
RA
Tielman
. “AIDS in The Netherlands: The Effects of Several Years of Campaigning.” International Journal of STD & AIDS
1(4), (July 1990): 268–75. 10.1177/095646249000100408
2088537

Wang, Ruey Hsia
, 
Min Tao
Hsu
, and 
Hsiu Hung
Wang
. 2004. “Potential Factors Associated with Contraceptive Intention among Adolescent Males in Taiwan.” Kaohsiung Journal of Medical Sciences
20(3): 115–123. 10.1016/s1607-551x(09)70094-4.15124895PMC11917642

Wang, Ruey‐Hsia
, 
Chung‐Ping
Cheng
, and 
Fan‐Hao
Chou
. 2008. “A Causal Model of Contraceptive Intention and Its Gender Comparison Among Taiwanese Sexually Inexperienced Adolescents.” Journal of Clinical Nursing
17(7): 930–9. 10.1111/j.1365-2702.2007.02088.x.18321290

Westhuizen
van der, N.
, and 
G.
Hanekom
. 2016. “Patient Knowledge and Intention to Use the Intrauterine Contraceptive Device (IUCD) at a Tertiary‐Level Hospital.” South African Journal of Obstetrics and Gynaecology
22(2): 42–46. 10.7196/SAJOG.2016.v22i2.1048.

Williams, M.
, 
A.
Bowen
, 
M.
Ross
, 
S.
Timpson
, 
U.
Pallonen
, and 
C.
Amos
. 2008. “An Investigation of a Personal Norm of Condom‐Use Responsibility among African American Crack Cocaine Smokers.” AIDS Care ‐ Psychological and Socio‐Medical Aspects of AIDS/HIV
20(2): 218–227. 10.1080/09540120701561288.PMC286026818293133

Wuni, Caroline
, 
Cornelius A.
Turpin
, and 
Edward T.
Dassah
. 2017. “Determinants of Contraceptive Use and Future Contraceptive Intentions of Women Attending Child Welfare Clinics in Urban Ghana.” BMC Public Health
18(1): 1–8. 10.1186/s12889-017-4641-9.28764670PMC5539629

Yan, Jin
, 
Joseph T.F.
Lau
, 
Hi Yi
Tsui
, 
Jing
Gu
, and 
Zixin
Wang
. 2012. “Prevalence and Factors Associated with Condom Use among Chinese Monogamous Female Patients with Sexually Transmitted Infection in Hong Kong.” Journal of Sexual Medicine
9(12): 3009–3017. 10.1111/j.1743-6109.2012.02945.x.23035945

Zimmers, E
, 
G
Privette
, 
R H
Lowe
, and 
F
Chappa
. 1999. “Increasing Use of the Female Condom through Video Instruction.” Perceptual and Motor Skills
88(3, Pt 2): 1071–1077. 10.2466/pms.1999.88.3c.1071.10485084

Zavier, A. J. Francis
, and 
Shireen J.
Jejeebhoy
. 2015. Contraceptive use and intensions among unmarried and married young women undergoing abortion in Bihar and Jharkhand, India. Asia‐Pacific Population Journal
30(1): 51–70.

**TABLE A4:** ITU items in the DHS questionnaire over time

DHS Model Questionnaire with Commentary – Phase 1 (1984–1989) (English) ITU in contraception sections	Pregnant women, past users, and never users of FP asked about their intentions to use (with the distinction between use in the near future and later use) and preferred method. The data provide an indication of future demand for services. The distinction b/w the near and the more distant future is to avoid the respondents misunderstanding the time references.	Do you intend to use a method to avoid pregnancy at any time in the future? Which method would you use? Do you intend to use (preferred method) in the next 12 months
DHS Model Questionnaire with Commentary – Phase 2 (1988–1993) (English)	As above	Do you intend to use a method to avoid pregnancy at any time in the future? Do you intend to use a method in the next 12 months? When you use a method, which method would you prefer to use?
DHS Model Questionnaire with Commentary – Phase 3 (1992–1997) (English) ITU moved from the contraception section to fertility preferences	As above, with the addition “ Such data provides an indication of the future demand for services” ALSO “In previous, DHS questionnaires were located in Section 3. They have been relocated here so that the contraceptive intentions will be closer to reproductive intentions”	Do you think you will use a method to avoid pregnancy within the next 12 months? Do you think you will use a method to avoid pregnancy at any time in the future? Which method would you prefer to use?
DHS Model Questionnaire with Commentary – Phase 4 (1997–2003) (English) ITU in fertility preferences	Women who are not currently using a method are asked whether they intend to use one in the future. There is strong evidence that such intentions are highly predictive of future use.	Do you intend to use a method to avoid pregnancy at any time in the future? Which method would you use?
DHS Model Questionnaire – Phase 5 (2003–2008) (English)		Do you think you will use a contraceptive method to delay or avoid pregnancy at any time in the future? Which contraceptive method would you prefer to use? (1)
DHS Model Questionnaire – Phase 6 (2008–2013) (English, French)		Do you think you will use a contraceptive method to delay or avoid pregnancy at any time in the future?
DHS Model Questionnaire – Phase 7 (English, French)		Do you think you will use a contraceptive method to delay or avoid pregnancy at any time in the future?
DHS Model Questionnaire – Phase 8 (English)		Do you think you will use a contraceptive method to delay or avoid pregnancy at any time in the future?

## Footnotes

1 Unmet need is defined as the number or percentage of women currently married or in union who are fecund and who desire to either terminate or postpone childbearing, but who are not currently using a contraceptive method (Measure Evaluation 2020).

2 See Moreau et al. (2019), Peterson et.al. (2013), Darroch and Singh (2013), and Alkema et al. (2013).

3 Collecting data about women's intention to use contraception has antecedents dating back to the World Fertility Survey in the 1970s.

4 Raine et.al. (2011) suggest that we need further research to determine how best to accurately assess contraceptive intention in the clinical setting and support it through counseling interventions.

5 Cohen's (1992) meta‐analysis found a medium‐to‐large magnitude effect associated with the impact of implementation intention formation on goal attainment.
